# Asparagine protects pericentral hepatocytes during acute liver injury

**DOI:** 10.1172/JCI163508

**Published:** 2023-04-03

**Authors:** Yu Sun, Hadrien Demagny, Adrien Faure, Francesca Pontanari, Antoine Jalil, Nadia Bresciani, Ece Yildiz, Melanie Korbelius, Alessia Perino, Kristina Schoonjans

**Affiliations:** Institute of Bioengineering, Ecole Polytechnique Fédérale de Lausanne, Lausanne, Switzerland.

**Keywords:** Hepatology, Metabolism, Amino acid metabolism, Cell stress

## Abstract

The nonessential amino acid asparagine can only be synthesized de novo by the enzymatic activity of asparagine synthetase (ASNS). While ASNS and asparagine have been implicated in the response to numerous metabolic stressors in cultured cells, the in vivo relevance of this enzyme in stress-related pathways remains unexplored. Here, we found ASNS to be expressed in pericentral hepatocytes, a population of hepatic cells specialized in xenobiotic detoxification. ASNS expression was strongly enhanced in 2 models of acute liver injury: carbon tetrachloride (CCl_4_) and acetaminophen. We found that mice with hepatocyte-specific *Asns* deletion were more prone to pericentral liver damage than their control littermates after toxin exposure. This phenotype could be reverted by i.v. administration of asparagine. Unexpectedly, the stress-induced upregulation of ASNS involved an ATF4-independent, noncanonical pathway mediated by the nuclear receptor, liver receptor homolog 1 (LRH-1; NR5A2). Altogether, our data indicate that the induction of the asparagine-producing enzyme ASNS acts as an adaptive mechanism to constrain the necrotic wave that follows toxin administration and provide proof of concept that i.v. delivery of asparagine can dampen hepatotoxin-induced pericentral hepatocellular death.

## Graphical abstract

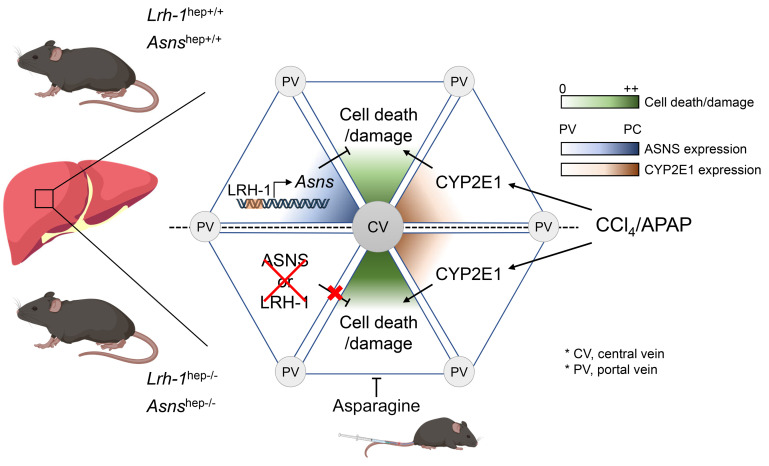

## Introduction

The liver is a highly structured tissue where oxygen-rich blood enters the hepatic lobule at peripheral portal triads and drains out through the central vein (reviewed in ref. 1). As blood flows, oxygen, nutrients, and hormones are taken up and metabolized by hepatocytes that actively shape their microenvironment and create a gradient along the lobular axis. In turn, this gradient, together with local morphogens, shapes the cellular identity and subsequent functional heterogeneity of cells within the lobule. Consequently, hepatic metabolic functions are nonuniformly distributed along the lobular axis, a phenomenon called liver zonation (reviewed in ref. 1). Energy-demanding functions, such as protein synthesis and secretion or de novo glucose production, are assigned to the periportal layers, where oxygen from arterial blood is abundant. Mid-lobular hepatocytes, on the other hand, specialize in the secretion of iron-regulating hormones, whereas pericentral hepatocytes preferentially engage in xenobiotic metabolism, bile acid biosynthesis, and glycolysis (reviewed in ref. 2).

Acute liver failure, characterized by sudden and severe hepatic injury, is a life-threatening condition (3). Drug-induced hepatotoxicity is the leading cause of acute liver failure in North American and European countries (3). Liver transplantation remains the most effective treatment for acute liver failure, but the lack of donor organs limits its availability. The survival rate of acute liver failure has stagnated in recent years, and novel treatment options are urgently needed to improve the overall outcome of patients (4, 5). Liver zonation also has a role in this process, as it can explain zonated damage during acute liver injury. The zonated nature of xenobiotic metabolism, for instance, is responsible for the observed pericentral damage upon drug overdose. This is due to the accumulation of toxic intermediates in the hepatocytes expressing the detoxification enzymes, including cytochrome P450 2E1 (CYP2E1) and cytochrome P450 1A2 (CYP1A2) (6, 7), and is exemplified in the carbon tetrachloride (CCl_4_) murine model of liver injury (8, 9). Although molecular CCl_4_ is not toxic, hepatotoxicity develops following its metabolic activation by CYP2E1 enzymes and the formation of the highly reactive trichloromethyl (CCl_3_) radicals. This highly reactive metabolite triggers oxidative damage to proteins, DNA, and lipids in pericentral CYP2E1-positive hepatocytes and culminates in liver injury and cell death (10).

Hepatic glutamine metabolism represents another highly zonated process where glutamine synthesis occurs through the catalytic action of glutamate-ammonia ligase (GLUL, also known as glutamine synthetase, [GS]), an enzyme whose expression is confined to a layer of hepatocytes surrounding the central veins, known as scavenger cells (reviewed in ref. 11). While glutamine synthesis from glutamate appears to be an efficient and required mechanism to capture ammonia (12), the fate of glutamine synthesized at high rates in the pericentral zone is unclear. In this study, we demonstrate that a pericentral enzyme, asparagine synthetase (ASNS), colocalizes with GLUL and converts its main product, glutamine, into asparagine. We show that during acute liver injury, pericentrally expressed ASNS is highly induced through the enhanced recruitment of the nuclear receptor liver receptor homolog 1 (LRH-1; NR5A2) to its promoter region. This transcriptional process is part of a noncanonical adaptive mechanism to protect pericentral hepatocytes from cellular death and can be therapeutically leveraged through i.v. delivery of asparagine, one of the enzymatic end products of ASNS.

## Results

### ASNS is expressed in GLUL^+^ pericentral hepatocytes and induced during acute liver injury.

**Figure 1:**
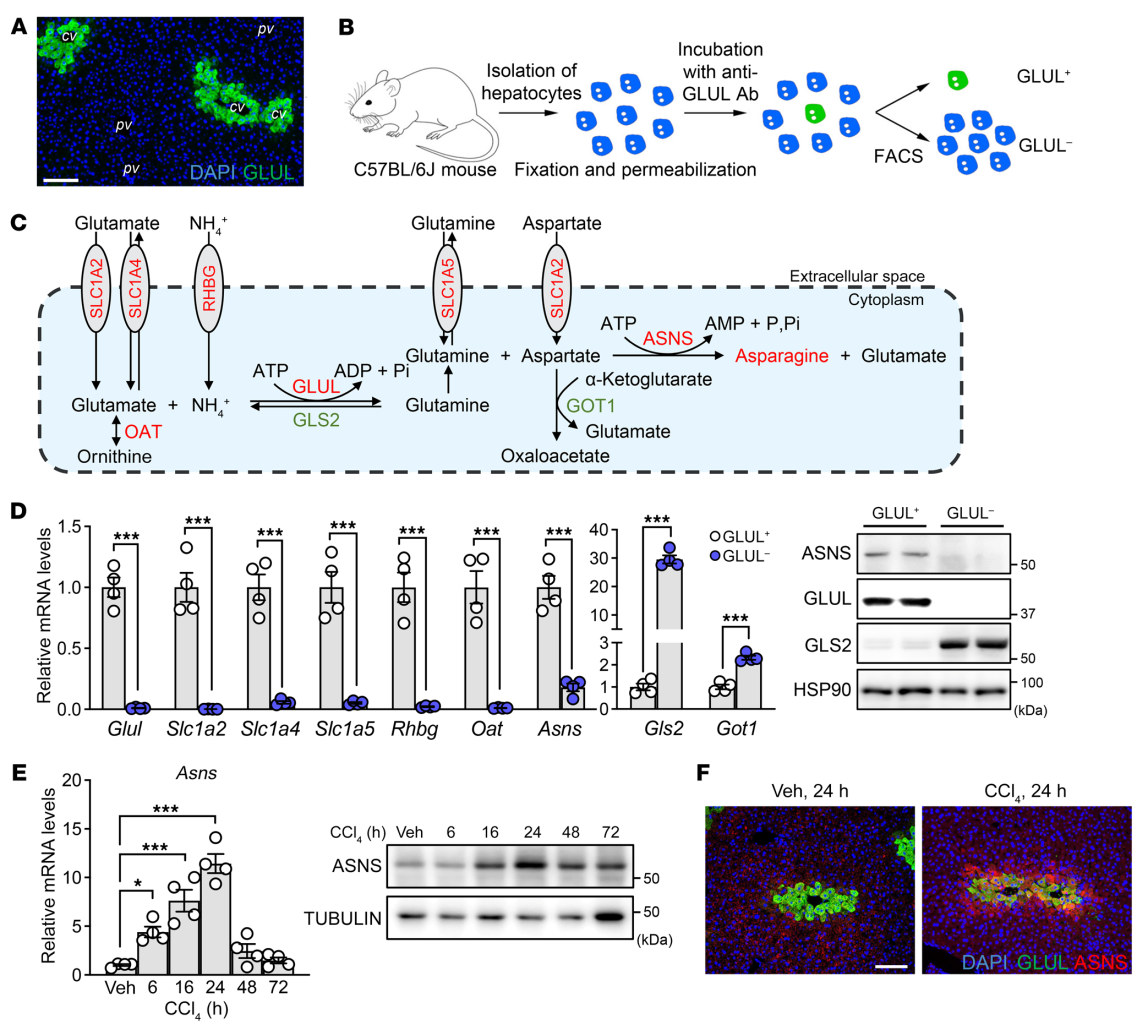
ASNS is expressed in GLUL^+^ pericentral hepatocytes and induced upon CCl_4_ treatment. (**A**) Immunofluorescent staining for GLUL and DAPI (nuclei, blue) in liver of a C57BL/6J mouse. cv, central vein; pv, portal vein. Scale bar: 100 μm. (**B**) Workflow of GLUL^+^ and GLUL^–^ hepatocytes isolation. FACS, fluorescence-activated cell sorting; Ab, antibody. (**C**) Scheme of glutamine metabolism in liver. (**D**) mRNA expression and protein levels of glutamine-related transporters and enzymes in sorted GLUL^+^ and GLUL^–^ hepatocytes. *n* = 4 animals for each group. (**E**) mRNA and western blotting analyses of total liver lysates from C57BL/6J mice collected at indicated time points after CCl_4_ treatment. *n* = 4 animals for each group. Veh, vehicle. (**F**) Immunofluorescent costaining for ASNS and GLUL with DAPI in livers treated with or without CCl_4_ for 24 hours. Scale bar: 100 μm. Error bars denote SEM. Statistical analysis was performed by unpaired *t* test (**D**) and 1-way ANOVA followed by Bonferroni’s posthoc test (**E**). *P < 0.05; ***P < 0.001.

The synthesis of glutamine in the liver is supported by the enzyme GLUL, whose expression is confined to hepatocytes surrounding the central veins (Figure 1A). To gain insights into the metabolism of this highly specialized population of GLUL^+^ hepatocytes, we developed a fluorescence-activated cell sorting–based (FACS-based) protocol to separate GLUL^+^ and GLUL^–^ hepatocytes from the livers of 10-to-12 week-old male C57BL/6J mice (Figure 1B and Supplemental Figure 1A; supplemental material available online with this article; https://doi.org/10.1172/JCI163508DS1). RNA and protein analyses from these FACS-sorted cells revealed that GLUL^+^ hepatocytes express several enzymes and transporters required for optimal glutamine production, such as the ammonium transporter rhesus type glycoprotein B (RHBG), the glutamate/aspartate transporter SLC1A2, the glutamate transporter SLC1A4, the glutamine transporter SLC1A5, and ornithine aminotransferase (OAT) (Figure 1, C and D), as previously described (13–16). We then asked what could be the fate of newly synthesized pericentral glutamine. Glutamine can be used to fuel and replenish intermediates of the tricarboxylic acid (TCA) cycle through the action of glutaminases (17). There are 2 glutaminase genes — *Gls1* and *Gls2* — but hepatic tissues solely express the liver-specific glutaminase *Gls2* (Supplemental Figure 1B) (18), which is predominantly expressed in GLUL^–^ hepatocytes (Figure 1D), suggesting that glutamine produced in pericentral zones is not used to fuel the TCA cycle. Glutamine can also serve as a nitrogen donor for asparagine synthesis via ASNS (Figure 1C). Further examination revealed that GLUL^+^ hepatocytes also expressed ASNS, both at the gene and protein levels (Figure 1D), demonstrating that hepatic asparagine de novo synthesis is a zonated process coinciding with glutamine metabolism in pericentral hepatocytes. Interestingly, GLUL^+^; ASNS^+^ hepatocytes were also highly enriched with *Slc1a2* (Figure 1D), the high-affinity amino acid transporter for aspartate (Figure 1C) (19), the other substrate of ASNS. Taken together, these data demonstrate that pericentral hepatocytes express the required set of enzymes and transporters to sustain asparagine synthesis.

In nonhepatic cells, ASNS expression is known to be induced in vitro by various metabolic stressors, such as glucose starvation (20), amino acid deprivation (21, 22), ER stress (23), and mitochondrial stress (24, 25). Altogether, ASNS levels are elevated in cultured cells when survival is compromised, and its expression has been consistently shown to counteract cell death. Because of their oxygen-deprived environment and exposure to toxic intermediates during xenobiotic detoxification, pericentral hepatocytes are particularly vulnerable to injury (26–28). To determine if ASNS expression alters under conditions that challenge the pericentral zones, we subjected C57BL/6J mice to a CCl_4_ model of acute liver injury (8, 9). CCl_4_ induces cell death and liver injury, specifically in pericentral hepatocytes, as a result of its conversion into toxic free radical metabolites catalyzed by the pericentrally zonated enzyme CYP2E1 (Supplemental Figure 1C) (29). As expected, CCl_4_ administration increased alanine aminotransferase (ALT) serum levels (Supplemental Figure 1D), indicating liver damage. Histological analysis revealed cellular vacuolization, cell swelling, and nuclear disintegration around central veins (Supplemental Figure 1E). TUNEL staining further confirmed that CCl_4_-induced cell death was confined to the pericentral zone (Supplemental Figure 1E). Of interest, ASNS mRNA and protein levels became massively induced after CCl_4_ administration, the former reaching its peak 24 hours after treatment while the latter remained high 48–72 hours after toxin injection (Figure 1E). Immunofluorescence staining confirmed that ASNS induction was confined to the liver’s pericentral, injured zone (Figure 1F). Our results demonstrate that induction of ASNS, a well-known prosurvival protein, is a common response of pericentral hepatocytes to drug-induced liver injury.

### ASNS protects pericentral hepatocytes from cell death during CCl_4_-induced acute liver injury.

**Figure 2:**
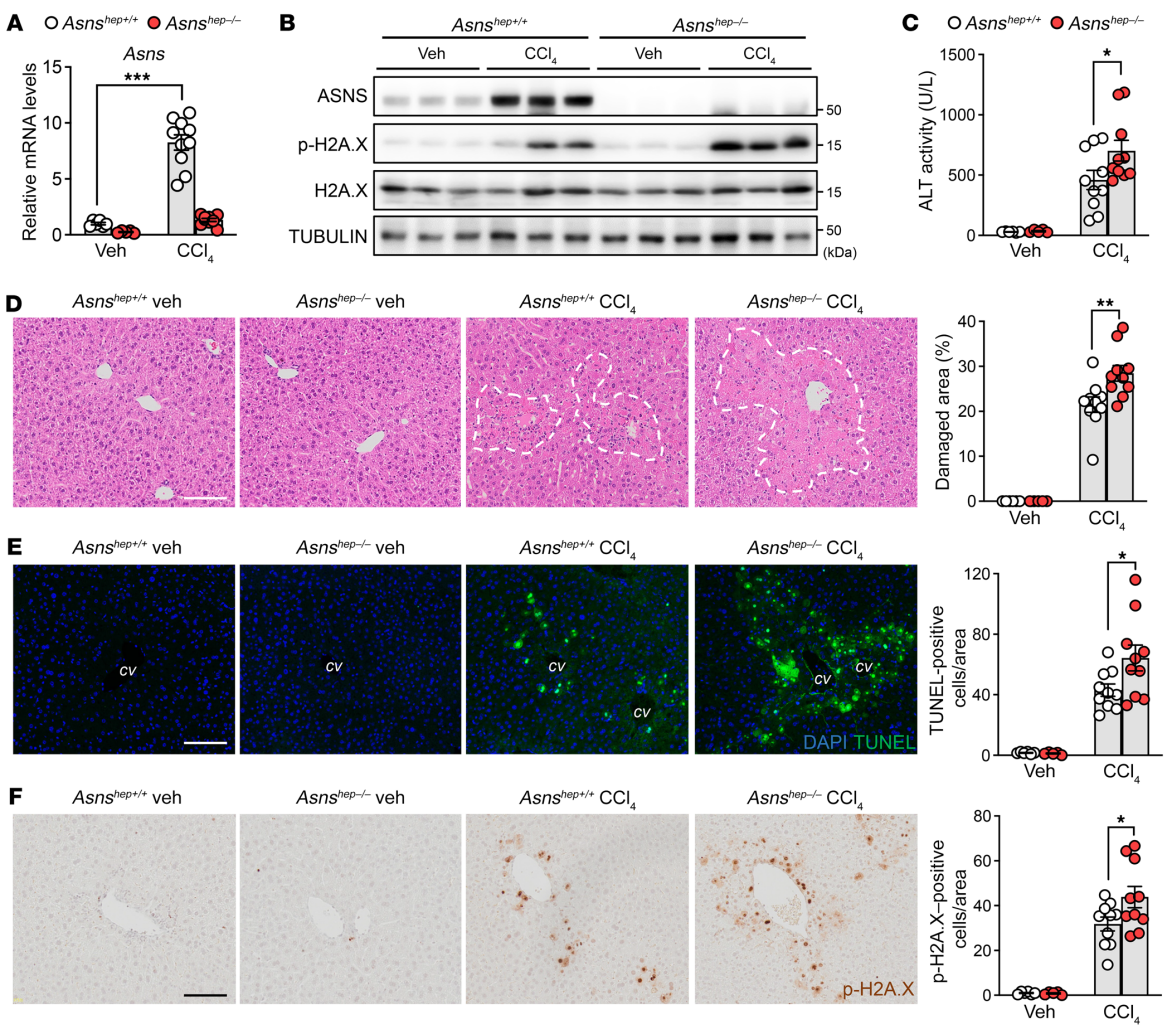
Loss of ASNS leads to enhanced CCl_4_-induced acute liver injury. (**A** and **B**) mRNA and Western blotting analyses of *Asns^hep+/+^* and *Asns^hep–/–^* mice treated with corn oil (veh) or CCl_4_ for 24 hours. *n* = 6 (*Asns^hep+/+^* veh); *n* = 5 (*Asns^hep–/–^* veh); and *n* = 10 (all other groups). (**C**) ALT activity in serum from vehicle or CCl_4_-treated mice in (**A**). (**D**–**F**) Representative images of H&E staining (**D**), TUNEL assay (**E**) and immunohistochemistry analysis of phospho-H2A.X (p-H2A.X) (**F**) in livers from (**A**). Damaged areas are outlined in white lines (**D**). Scale bar: 100 μm. cv, central vein. Quantification results are indicated on the right. Error bars denote SEM. Statistical analysis was performed by 2-way ANOVA followed by Bonferroni’s posthoc test (**A**, **C**–**F**). *P < 0.05; **P < 0.01; ***P < 0.001.

To investigate the physiological function of ASNS during acute liver injury, we conditionally deleted *Asns* in hepatocytes by crossing *Asns^lox/lox^* mice with albumin-Cre transgenic animals, which specifically express the Cre recombinase in hepatocytes (Supplemental Figure 2, A–C). Mice with hepatocyte-specific Asns deletion (*Asns^hep–/–^*) were born at the expected Mendelian frequency and did not display apparent phenotypes compared with control littermates (*Asns^hep+/+^*) under unchallenged conditions (data not shown). We then assessed the role of ASNS during acute liver injury by administering CCl_4_. The induction of ASNS, both at the mRNA (Figure 2A and Supplemental Figure 2D) and protein (Figure 2B) levels, was completely blunted in *Asns^hep–/–^* livers upon CCl_4_ challenge, confirming that its upregulation occurs in the hepatocyte lineage during acute liver injury. ALT levels were enhanced in the serum of *Asns^hep–/–^* mice, indicating more severe liver injury upon CCl_4_ in *Asns*-deficient animals (Figure 2C and Supplemental Figure 2E). At the histological level, the distinctive centrilobular damage induced after CCl_4_ was also enhanced in CCl_4_-treated *Asns^hep–/–^* mice (Figure 2D). Furthermore, the loss of ASNS in hepatocytes markedly increased the number of CCl_4_-induced cells positive for TUNEL staining (Figure 2E). These cells were also positive for phosphorylated histone H2A.X (p-H2A.X), a specific marker of DNA double-strand breaks (30). We observed more p-H2A.X-positive signals in *Asns^hep–/–^* livers than in control littermates after the CCl_4_ challenge (Figure 2, B and F), confirming the enhanced susceptibility to necrosis of ASNS-depleted pericentral hepatocytes.

After being metabolized by CYP2E1, CCl_4_ is turned into CCl_3_ radical, a highly reactive metabolite known to trigger lipid peroxidation and, eventually, liver damage (Supplemental Figure 1C) (31). As previously reported, *Cyp2e1* RNA levels were downregulated after CCl_4_ injection (Supplemental Figure 2F) (32). Importantly, loss of ASNS had no impact on *Cyp2e1* levels in unchallenged or challenged conditions (Supplemental Figure 2F), discarding the possibility that hepatic ASNS affects the detoxification machinery. In line with this, 4-hydroxynonenal (4-HNE) staining, used as a readout for lipid peroxidation (33), was enhanced in the livers of CCl_4_-treated animals, but not affected by the presence or absence of ASNS (Supplemental Figure 2G). Notably, the proliferation marker *Ki67* was similarly induced in *Asns^hep–/–^* and *Asns^hep+/+^* mice 48 hours after CCl_4_ injection (Supplemental Figure 2H). In contrast, *Asns* induction peaked 24 hours after toxin administration, suggesting that ASNS does not affect liver regeneration and that its induction upon CCl4 is not limited to regenerating cells. Together, our results reveal that ASNS acts downstream of CCl_3_ production and lipid peroxidation and is required to constrain the widespread pericentral necrotic wave that follows CCl_4_ injection.

### ASNS protects against APAP-induced acute liver injury, and its expression is induced by various liver stressors.

**Figure 3:**
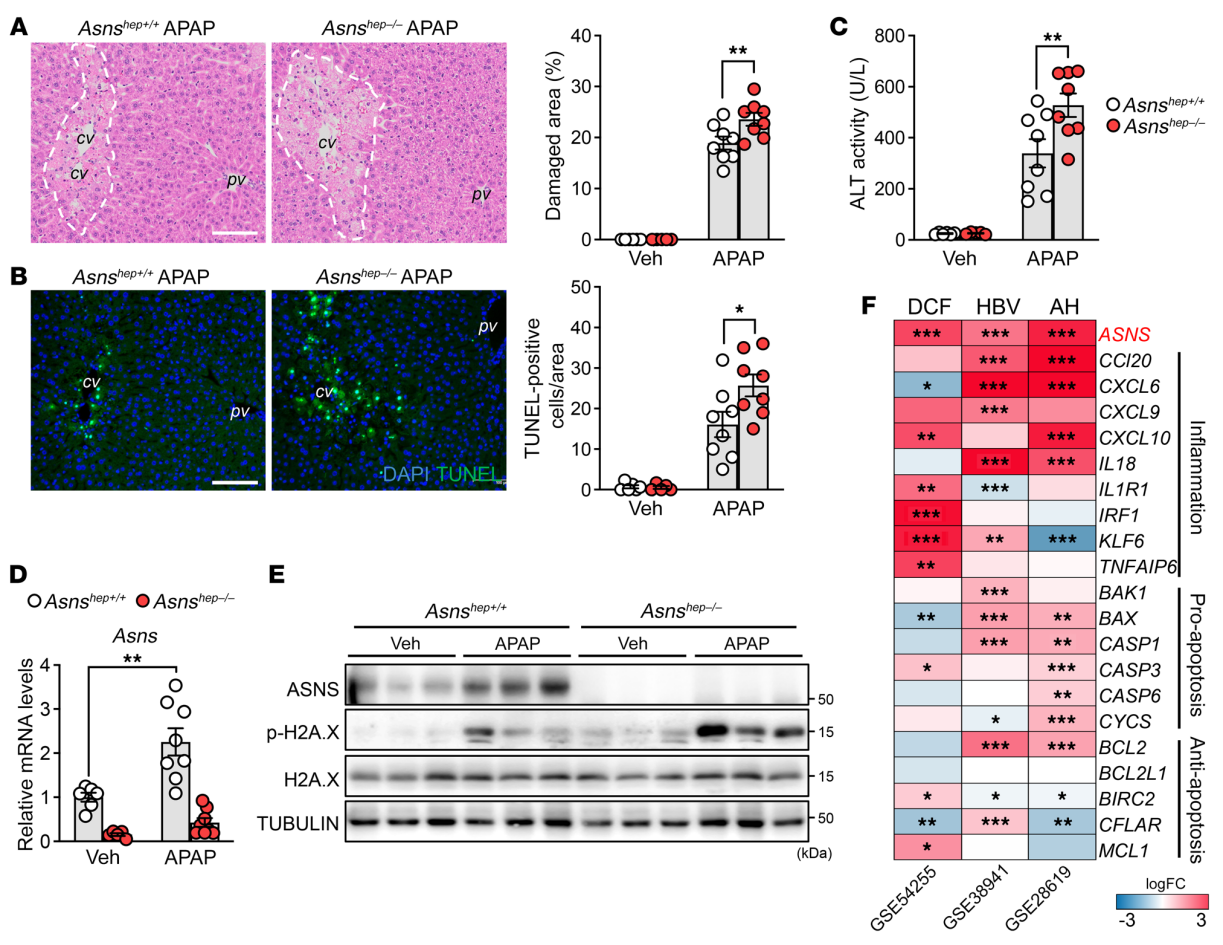
ASNS protects against APAP-induced acute liver injury and its expression is induced by various liver stressors. (**A** and **B**) H&E staining (**A**) and TUNEL assay (**B**) in livers of *Asns^hep+/+^* and *Asns^hep–/–^* mice treated with PBS (veh) or APAP for 24 hours. *n* = 6 (*Asns^hep+/+^* veh); *n* = 5 (*Asns^hep–/–^* veh); and *n* = 8 (all other groups). Scale bar: 100 μm. (**C**) Serum ALT activity in mice from (**A**). (**D** and **E**) mRNA and protein analyses of total cell lysates in livers from (**A**). (**F**) Heatmap showing logFC expression of genes in each microarray between treated versus untreated human liver slices (GSE54255, *n* = 5 for each group), or diseased versus healthy human livers (GSE38941, *n* = 17 for HBV and *n* = 10 for normal livers; and GSE28619, *n* = 15 for AH and *n* = 7 for normal livers). *P* values are adjusted by Benjamini & Hochberg. DCF, diclofenac; HBV, hepatitis B virus; AH, alcoholic hepatitis. Error bars denote SEM. Statistical analysis was performed by 2-way ANOVA followed by Bonferroni’s posthoc test (**A**–**D**). *P < 0.05; **P < 0.01; ***P < 0.001.

Acetaminophen (APAP) is a widely used analgesic, and its overdose accounts for nearly half of all drug-induced hepatotoxicity cases in Western countries (3, 34). As a result of its conversion into the toxic reactive metabolite NAPQI, catalyzed by the pericentral enzyme CYP2E1, APAP also induces necrosis, specifically in CYP2E1-positive pericentral hepatocytes. We injected mice with a low, sub-lethal dose (300 mg/kg) to cause damage in the pericentral zone (35). Loss of ASNS in hepatocytes markedly enlarged the APAP-induced pericentral damage irrespective of the nutritional status (Figure 3A and Supplemental Figure 3B) and increased the number of TUNEL-positive cells (Figure 3B). In both fed and fasted conditions, ALT levels were increased in APAP-treated *Asns^hep–/–^* mice (Figure 3C and Supplemental Figure 3A), indicating a more pronounced susceptibility sto liver injury in *Asns*-deficient animals. mRNA and protein analyses revealed that ASNS expression was strongly induced after APAP administration (Figure 3, D and E and Supplemental Figure 3C) and that the blunted ASNS induction in *Asns^hep–/–^* mice exacerbated cell death after APAP exposure (Figure 3E). Moreover, data mining into human liver data sets revealed that *ASNS* expression was highly induced in diclofenac-treated human liver slices (GSE54255) and livers of patients with HBV-associated acute liver failure (GSE38941) or alcoholic hepatitis (GSE28619), indicating that ASNS may play an essential role in acute and chronic liver conditions triggered by toxins or viral infections (Figure 3F).

### The nuclear receptor LRH-1 controls expression of ASNS in pericentral hepatocytes.

**Figure 4:**
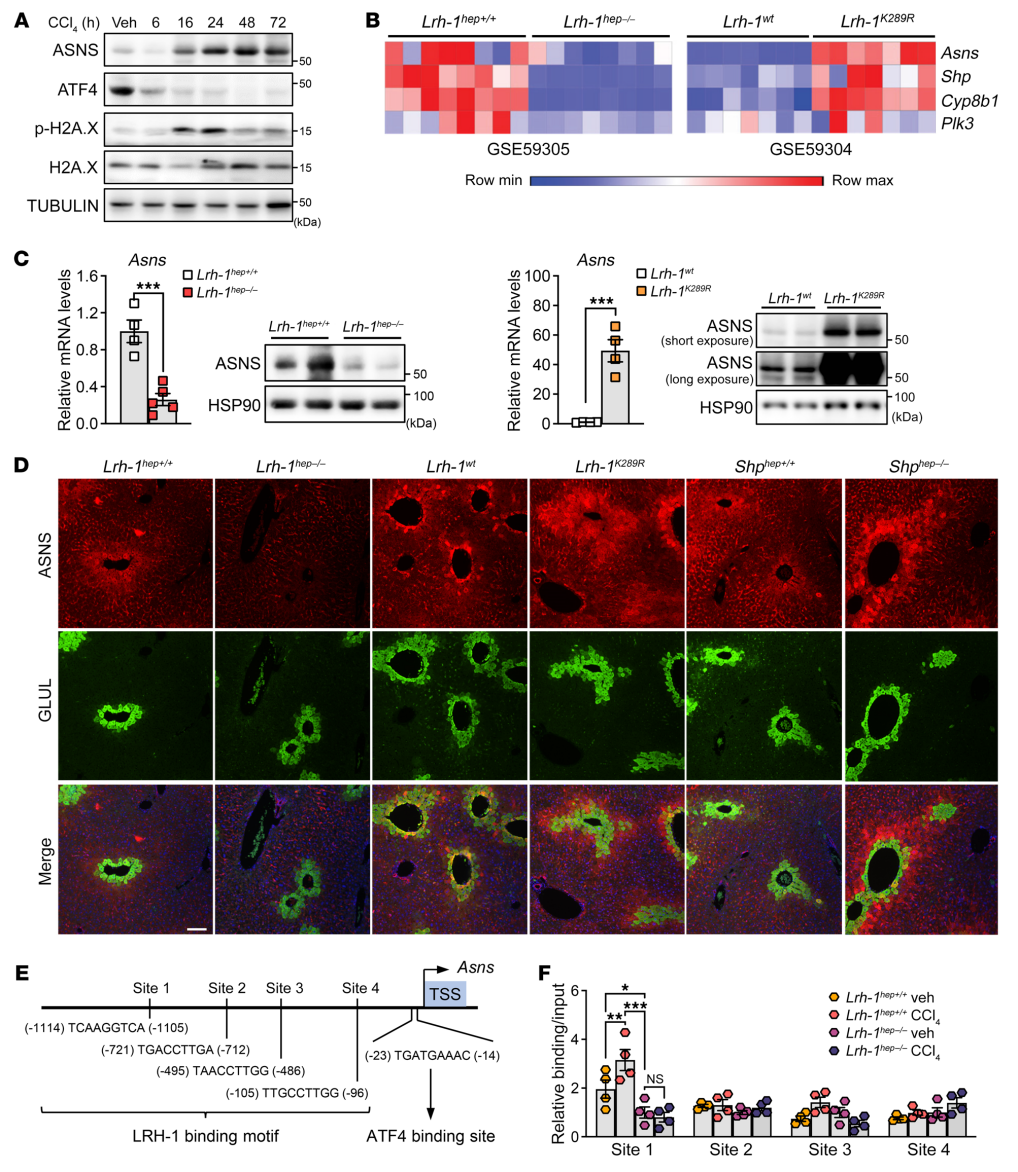
*Asns* is a direct LRH-1 targeted pericentral gene. (**A**) Western blotting analysis of total liver lysates from C57BL/6J mice collected at indicated time points after CCl_4_ treatment. (**B**) Heatmap showing the expression levels of *Asns* and known LRH-1 targets in publicly available data sets (GSE59305 and GSE59304). (**C**) mRNA and protein analyses of total cell lysates from livers of *Lrh-1^hep+/+^* and *Lrh-1^hep–/–^* mice, or *Lrh-1^wt^* and *Lrh-1^K289R^* mice. *n* = 4 (*Lrh-1^hep+/+^*, *Lrh-1^wt^*, and *Lrh-1^K289R^*) and *n* = 5 (*Lrh-1^hep–/–^*). (**D**) Representative images of immunofluorescent staining for ASNS and GLUL in livers from the indicated genetically modified mouse lines. Scale bar: 100 μm. (**E**) Transcription factor binding site analysis of mouse *Asns* promoter sequence showed 1 ATF4 binding site and 4 predicted LRH-1 binding sites. Numbers indicate distance from transcription start site (TSS). (**F**) Binding of LRH-1 to the 4 *Asns* promoter sites assessed by ChIP analysis using genomic DNA from livers of *Lrh-1^hep+/+^* and *Lrh-1^hep–/–^* mice treated with or without CCl_4_ for 24 hours. *n* = 4 animals for each group. Error bars denote SEM. Statistical analysis was performed by unpaired *t* test (**C**) and 2-way ANOVA followed by Bonferroni’s post-hoc test (**F**). *P < 0.05; **P < 0.01; ***P < 0.001.

We next sought to determine the molecular mechanism responsible for the induction of *Asns* during acute liver injury by administrating CCl_4_, the most potent inducer of ASNS (Figure 1E and 2A). Intracellular imbalance in amino acid composition activates the *Asns* gene through the amino acid response (36, 37). ER stress also increases *Asns* transcription through the protein kinase R-like ER kinase–eukaryotic initiation factor 2 (PERK-eIF2) arm of the unfolded protein response (UPR) (23). Both the AAR and UPR lead to increased synthesis of activating transcription factor 4 (ATF4), which binds to the C/EBP-ATF response element and induces *Asns* transcription. Therefore, ATF4 is considered a master regulator of *Asns* transcription (38). We first assessed hepatic ATF4 levels after CCl_4_ treatment. Consistent with previous reports (39), ATF4 levels remarkably decreased following CCl_4_ injection (Figure 4A), suggesting that *Asns* is induced through a different mechanism. We then interrogated *Asns* expression profiles of liver data sets (GSE59305 and GSE59304) to identify a putative regulator of hepatic *Asns*. *Asns* mRNA levels were blunted in LRH-1-depleted livers (18, 40) while being robustly upregulated in the livers of knockin mice carrying a selective gain-of-function point mutation in LRH-1 (*Lrh-1^K289R^*) mice (41–43) (Figure 4B). A similar pattern of regulation was observed for other well-described LRH-1 target genes, such as *Shp* (44), *Cyp8b1* (45), and *Plk3* (46) (Figure 4B). Quantitative real-time PCR (qRT-PCR) and immunoblotting confirmed reduced ASNS gene and protein expression in hepatocyte-specific *Lrh-1* knockout mice (*Lrh-1^hep–/–^*) and a strong upregulation of this gene in *Lrh-1* gain-of-function knockin mice (*Lrh-1^K289R^*, Figure 4C). Immunofluorescence analysis confirmed the disappearance of ASNS protein in the pericentral layer of *Lrh-1^hep–/–^* mice (Figure 4D). In *Lrh-1^K289R^* livers, ASNS protein levels were strongly increased but remained localized around the central veins (Figure 4D), demonstrating that this enzyme’s zonated pattern of expression was retained. To further reinforce the link between hepatic ASNS expression and LRH-1 activity, we analyzed the livers of mice carrying a liver-specific deletion of small heterodimer partner (SHP, NR0B2) (*Shp^hep–/–^*) (47). SHP is a unique member of the nuclear receptor superfamily that lacks a DNA binding domain and acts as a potent inhibitor of several nuclear receptors, in particular LRH-1 (43, 48, 49). ASNS levels were found to be strongly increased in the livers of *Shp^hep–/–^* mice while remaining pericentral (Figure 4D). Finally, we asked whether *Asns* was a direct transcriptional target of LRH-1. For this purpose, we analyzed genomic regions surrounding the *Asns* gene. We identified 4 potential binding sites with an LRH-1 consensus sequence (50) in the proximal 5′ regulatory sequence upstream of the transcription start site (–1200 bp to 0 bp) (Figure 4E). We then performed site-specific ChIP analysis to investigate whether LRH-1 was recruited to these sites in control and CCl_4_-treated *Lrh-1^hep+/+^* and *Lrh-1^hep–/–^* mice. Under unchallenged conditions, we found a weak but significant binding of LRH-1 to site 1 in the *Asns* promoter region, specifically in *Lrh-1^hep+/+^* mice (Figure 4F). This weak recruitment of LRH-1 is consistent with the relatively low basal levels of ASNS under unchallenged conditions (Figure 1F). However, the binding of LRH-1 to site 1 was enhanced after the CCl_4_ challenge (Figure 4F), suggesting that acute liver injury stimulates recruitment of LRH-1 to the *Asns* promoter. To reinforce these results, we cloned the mouse *Asns* promoter upstream of the luciferase gene and performed targeted mutagenesis combined with reporter assays. As shown in Supplemental Figure 4, the mouse *Asns* promoter responded very well to cotransfection with LRH-1 (Supplemental Figure 4). In this artificial in vitro system, all putative LRH-1 sites appeared to play some role in the induction of luciferase (Supplemental Figure 4). Taken together, our data demonstrate that *Asns* is a direct transcriptional target of LRH-1 in the liver and that this nuclear receptor mediates the upregulation of ASNS under challenging conditions.

### The LRH-1-ASNS axis protects pericentral hepatocytes from cell death during acute liver injury.

**Figure 5:**
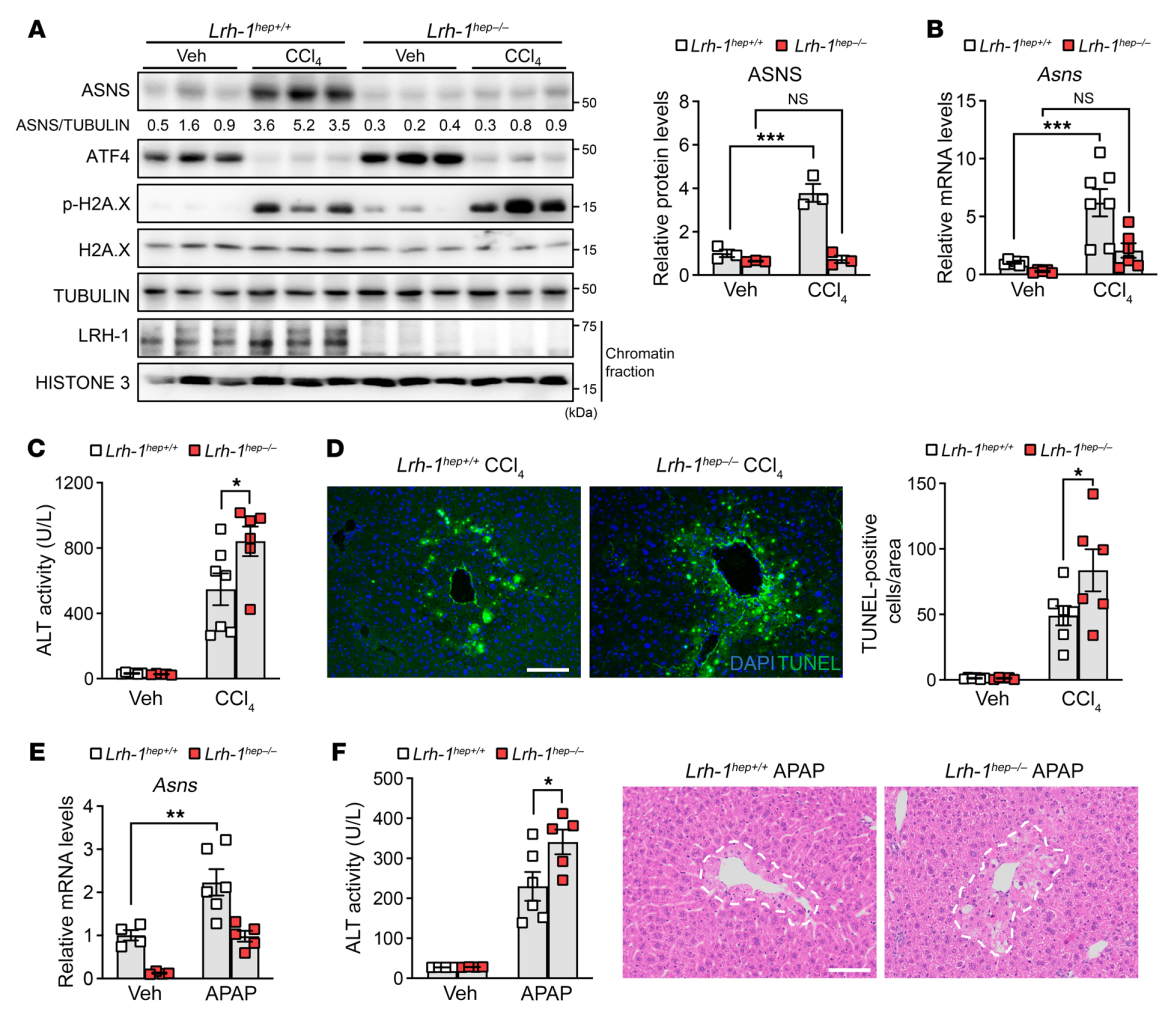
Loss of LRH-1 exacerbates acute liver injury triggered by CCl_4_ or APAP. (**A**) Western blotting analysis of total cell lysates or chromatin fractions from livers of *Lrh-1^hep+/+^* and *Lrh-1^hep–/–^* mice treated or untreated with CCl_4_ for 24 hours. Quantification of blotting analysis showing the relative levels of ASNS protein compared with the loading control TUBULIN. (**B**) mRNA expression levels of *Asns* in CCl_4_-treated livers from (**A**). *n* = 6 (*Lrh-1^hep+/+^* veh, *Lrh-1^hep–/–^* CCl_4_); *n* = 5 (*Lrh-1^hep–/–^* veh); and *n* = 7 (*Lrh-1^hep+/+^* CCl_4_). (**C**) Serum ALT activity in mice from (**B**). (**D**) TUNEL assay in livers from (**B**). Scale bar: 100 μm. (**E**) mRNA analysis of livers from *Lrh-1^hep+/+^* and *Lrh-1^hep–/–^* mice treated with or without APAP for 24 hours. *n* = 4 (*Lrh-1^hep+/+^* veh, *Lrh-1^hep–/–^* veh); *n* = 6 (*Lrh-1^hep+/+^* APAP); and *n* = 5 (*Lrh-1^hep–/–^* APAP). (**F**) ALT activity in serum and H&E staining in livers from mice in (**E**). Scale bar: 100 μm. Error bars denote SEM. Statistical analysis was performed by 2-way ANOVA followed by Bonferroni’s posthoc test (**A**–**F**). *P < 0.05; **P < 0.01; ***P < 0.001.

To determine the importance of the newly identified LRH-1-ASNS axis during liver injury, we subjected *Lrh-1^hep–/–^* mice and control (*Lrh-1^hep+/+^*) littermates to a CCl_4_ protocol. The sharp rise in ASNS protein (Figure 5A) and transcript (Figure 5B) levels upon CCl_4_ challenge was completely blunted in *Lrh-1^hep–/–^* livers (Figure 5, A and B), demonstrating that LRH-1 is required for ASNS induction under challenging conditions. In line with data from *Asns^hep–/–^* mice, we found increased liver damage in *Lrh-1^hep–/–^* mice, as measured by ALT serum levels, TUNEL staining, and p-H2A.X staining and blotting (Figure 5, A, C, and D and Supplemental Figure 5). We then turned to the APAP model of liver injury and found that the upregulation of *Asns* upon APAP was partially blunted in *Lrh-1^hep–/–^* livers (Figure 5E). Similar to CCl_4_, APAP-induced ALT serum levels and centrilobular necrotic areas were significantly increased in *Lrh-1^hep–/–^* mice (Figure 5F), demonstrating that genetic deletion of *Lrh-1* phenocopies the loss of *Asns* and renders pericentral hepatocytes more susceptible to cell death in 2 preclinical models of liver injury.

**Figure 6:**
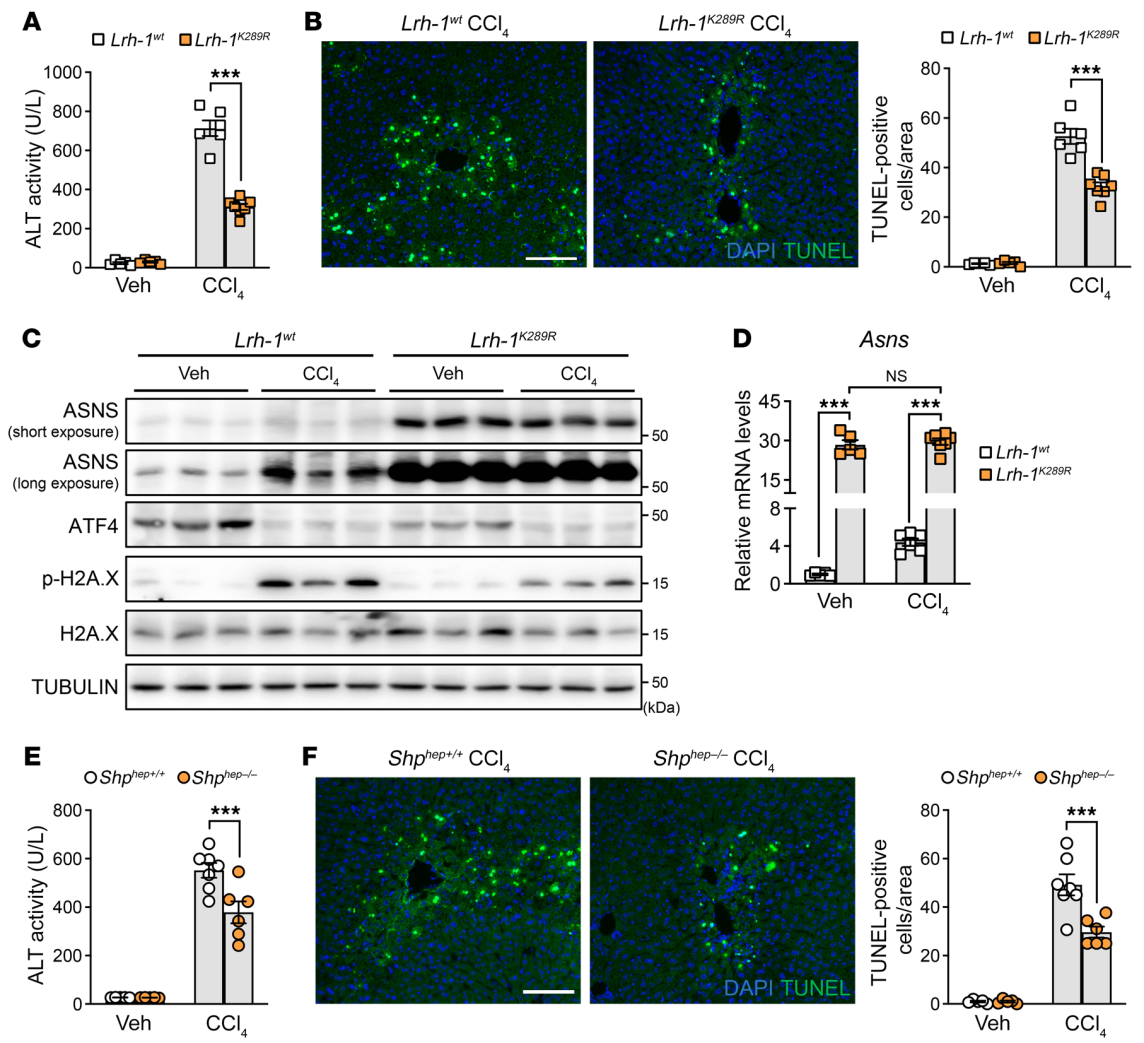
Activation of LRH-1 protects the mice against CCl_4_-induced acute liver injury. (**A**) Serum ALT activity of *Lrh-1^wt^* and *Lrh-1^K289R^* mice treated 24 hours with or without CCl_4_. *n* = 5 (*Lrh-1^wt^* veh, *Lrh-1^K289R^* veh); *n* = 6 (*Lrh-1^wt^* CCl_4_); and *n* = 7 (*Lrh-1^K289R^* CCl_4_). (**B**) Representative images and quantification results of TUNEL staining in livers from (**A**). (**C** and **D**) Western blotting and mRNA analyses of total liver lysates from (**A**). (**E**) Serum ALT activity of *Shp^hep+/+^* and *Shp^hep–/–^* mice treated with or without CCl_4_ for 24 hours. *n* = 5 (*Shp^hep+/+^* veh, *Shp^hep–/–^* veh); *n* = 7 (*Shp^hep+/+^* CCl_4_); and *n* = 6 (*Shp^hep–/–^* CCl_4_). (**F**) Representative images of TUNEL staining of livers from (**E**). Quantification results are indicated on the right. Scale bar: 100 μm (**B** and **F**). Error bars denote SEM. Statistical analysis was performed by 2-way ANOVA followed by Bonferroni’s posthoc test (**A**, **B**, and **D**–**F**). ***P < 0.001.

We then analyzed the livers of *Lrh-1^K289R^* knockin mice in which ASNS expression is constitutively high in the pericentral zone (Figure 4D). Liver damage was blunted when these animals were treated with CCl_4_, as evidenced by the reduced ALT serum levels (Figure 6A). Similarly, *Lrh-1^K289R^* pericentral hepatocytes, expressing higher levels of ASNS (Figure 4, C and D and Figure 6, C and D), were found to be more resilient to cell death upon CCl_4_ challenge, as shown by TUNEL staining(Figure 6B) and p-H2A.X staining and blotting (Figure 6C and Supplemental Figure 6A). To further reinforce the link between LRH-1 activity, ASNS, and prosurvival properties, we assessed the livers of *Shp^hep–/–^* mice. Western-blot (Supplemental Figure 6B) and qRT-PCR (Supplemental Figure 6C) experiments revealed much higher ASNS levels under basal conditions, confirming the immunofluorescence data (Figure 4D). These constitutively high ASNS levels could not be further boosted by CCl_4_ exposure (Supplemental Figure 6, B and C). Similar to the *Lrh-1^K289R^* mice, we found that *Shp^hep–/–^* mice were less affected by liver damage after CCl_4_ treatment (Figure 6E). SHP-depleted pericentral hepatocytes were also protected from CCl_4_-induced cell death (Figure 6F and Supplemental Figure 6B), further supporting that high levels of ASNS protect pericentral hepatocytes from the harmful effects of toxins during acute liver injury. Our results demonstrate that the prosurvival upregulation of ASNS is mediated through a novel pathway involving the nuclear receptor LRH-1. Genetic mutations known to boost LRH-1 activity strongly increase ASNS levels around the central veins and protect cells in this layer from cell death during acute liver injury.

### The cytoprotective actions of ASNS are mediated by asparagine.

**Figure 7:**
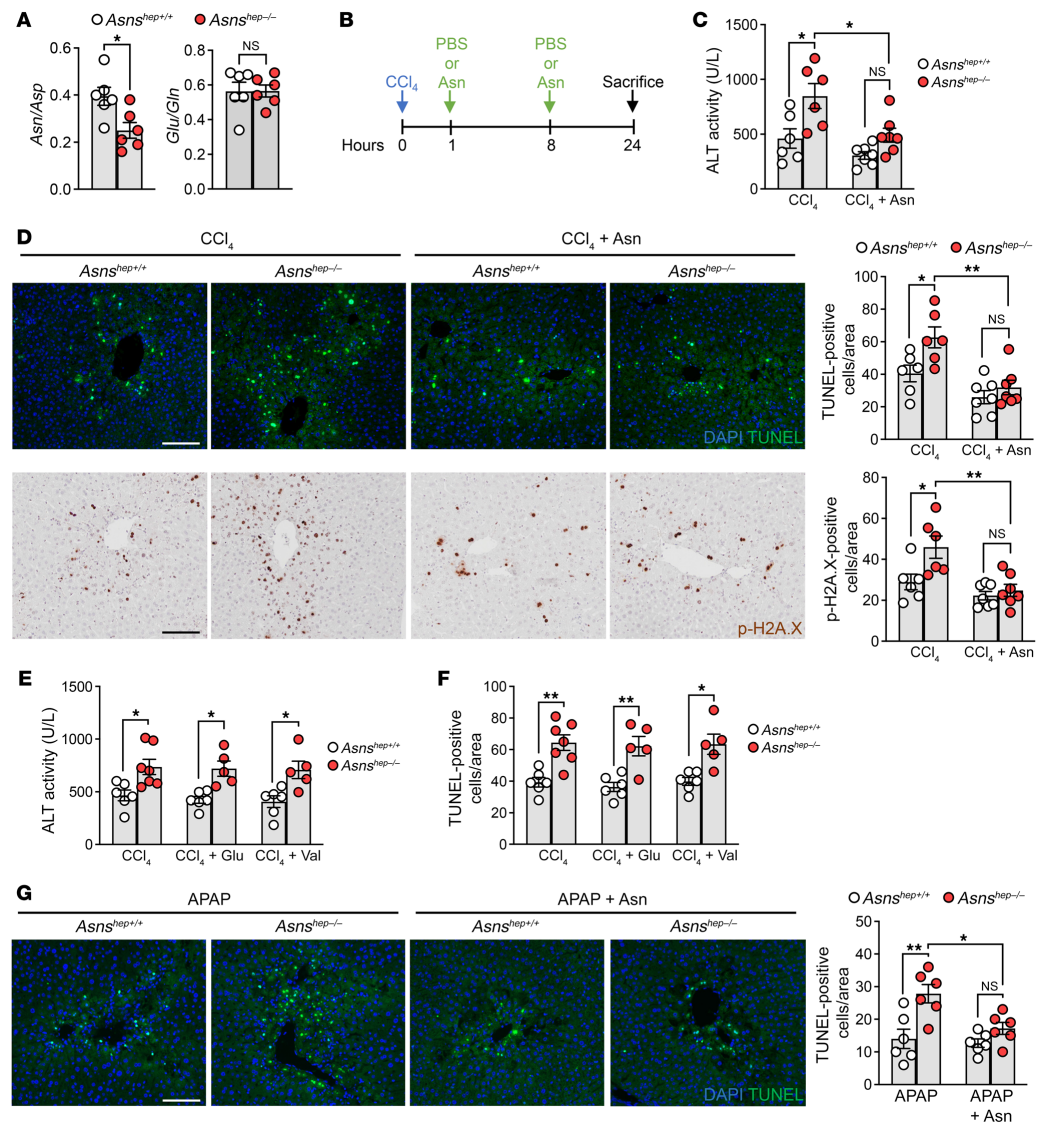
Asparagine treatment rescues *Asns* depletion-induced cell death and liver damage. (**A**) Asparagine (Asn) over aspartate (Asp) ratio and glutamate (Glu) over glutamine (Gln) ratio in livers of untreated *Asns^hep+/+^* and *Asns^hep–/–^* mice. *n* = 6 animals for each group. (**B**) Workflow of asparagine delivery upon CCl_4_ treatment. Mice were i.p. injected with CCl_4_ followed by 2 i.v. injections of 240 mg/kg asparagine (Asn) or PBS 1 hour and 8 hours later. (**C**) Serum ALT activity of *Asns^hep+/+^* and *Asns^hep–/–^* mice subjected to the treatment described in (**B**). *n* = 6 (CCl_4_ of *Asns^hep+/+^* and *Asns^hep–/–^*) and *n* = 7 (CCl_4_ + Asn of *Asns^hep+/+^* and *Asns^hep–/–^*). (**D**) Representative images of TUNEL assay and immunohistochemistry analysis of p-H2A.X in livers from (**C**). Scale bar: 100 μm. Quantification results are indicated on the right. (**E**–**F**) Serum ALT activity and quantification results of TUNEL staining in livers from CCl_4_-treated *Asns^hep+/+^* and *Asns^hep–/–^* mice, followed by i.v. injection of glutamate (Glu) or valine (Val). *n* = 6 (*Asns^hep+/+^* CCl_4_, CCl_4_ + Glu and CCl_4_ + Val); *n* = 7 (CCl_4_ of *Asns^hep–/–^*); and *n* = 5 (*Asns^hep–/–^* CCl_4_ + Glu and CCl_4_ + Val). (**G**) Representative images and quantification results of TUNEL assay in livers from APAP-treated *Asns^hep+/+^* and *Asns^hep–/–^* mice, followed by asparagine (Asn) i.v. injection. *n* = 6 animals for each group. Error bars denote SEM. Statistical analysis was performed by unpaired *t* test (**A**) and 2-way ANOVA followed by Bonferroni’s posthoc test (**C**–**G**). *P < 0.05; **P < 0.01.

The irreversible enzymatic activity of ASNS consumes glutamine and aspartate and produces 2 nonessential amino acids, glutamate and asparagine (51), the latter of which has been reported to counteract apoptosis in cultured cells (20–22, 52). Amino acid profiling using hydrophilic interaction chromatography–based (HILIC-based) high-resolution mass spectrometry (HRMS) (53) revealed that the ratio of glutamate over glutamine was unchanged in the livers of *Asns^hep–/–^* mice. In contrast, the ratio of asparagine over aspartate was significantly reduced in these animals (Figure 7A). Similarly, we found this same ratio to be decreased in *Lrh-1* loss-of-function mouse models (*Lrh-1^hep–/–^*, Supplemental Figure 7A) and strongly increased in *Lrh-1* gain-of-function mouse models with enhanced *Asns* expression (*Lrh-1^K289R^* and *Shp^hep–/–^*, Supplemental Figure 7A). Based on these findings, we posited that the lack of local asparagine production in ASNS-depleted pericentral hepatocytes could be responsible for their enhanced susceptibility to cell death. To test this hypothesis, we undertook a rescue experiment by providing both *Asns^hep+/+^* and *Asns^hep–/–^* mice an asparagine load shortly after the CCl_4_ injection. Mice received 2 tail-vein injections of asparagine dissolved in PBS at 240 mg/kg 1 hour and 8 hours after CCl_4_ administration (Figure 7B). Profiling of liver amino acids confirmed that i.v. injected asparagine reached the liver 30 minutes after administration (Supplemental Figure 7B). This bolus of asparagine effectively dampened and rescued liver damage in *Asns^hep–/–^* mice, as evidenced by the reduced ALT level (Figure 7C). In ASNS-depleted hepatocytes, asparagine reduced the number of necrotic pericentral hepatocytes after the CCl_4_ challenge (Figure 7D). Furthermore, the induction of ASNS after CCl_4_ injection was decreased slightly in asparagine-rescued mice (Supplemental Figure 7, C and D), suggesting that exogenously provided asparagine can overcome the need to locally produce prosurvival amino acids in pericentral cells. To confirm the unique role of asparagine in dampening cell death, we repeated the same protocol with glutamate, the other product of ASNS, and valine, an amino acid seemingly unrelated to ASNS. Both amino acids could not rescue the increased susceptibility to liver damage observed in *Asns^hep–/–^* mice (Figure 7, E and F and Supplemental Figure 7E), suggesting that asparagine exhibits unique cytoprotective properties. Finally, we attempted to rescue liver damage in APAP-treated *Asns^hep–/–^* mice. We applied the same protocol used in the CCl4 challenge experiment with 2 tail-vein injections of asparagine dissolved in PBS at 240 mg/kg 1 hour and 8 hours after APAP administration. In this model, again, asparagine could completely rescue the increased damage observed in ASNS-deficient animals (Figure 7G and Supplemental Figure 7F). Taken together, our results indicate that asparagine mediates the beneficial effect of ASNS induction during acute liver injury.

## Discussion

This study demonstrates that induction of ASNS in pericentral hepatocytes through a noncanonical, LRH-1-mediated mechanism dampens pericentral damage during acute liver injury. ASNS and asparagine have already been proposed to counteract cell death in cultured cells challenged by metabolic stressors such as glucose or glutamine starvation, ER stress, and mitochondrial insults (20–25, 52). The current study not only extends these in vitro data by showing that ASNS plays a protective role in preclinical models of acute liver damage but also suggests that the prosurvival function of ASNS is not limited to apoptosis, as acute liver injury following toxin exposure predominantly induces necrosis (27, 28). While our work with asparagine and glutamate supplementation strongly suggests that asparagine mediates the positive effects of ASNS upregulation during acute liver injury, we cannot exclude the possibility that a metabolic derivative of asparagine is the final effector in the regulation of this process. Further work is required to decipher the proper molecular mechanism linking asparagine to its cytoprotective effects. Our study also provides proof of concept that asparagine could act as a first line of defense to reverse drug-induced liver injuries. Notably, the liver is well-equipped with plasma membrane transporters involved in asparagine uptake, including transporters of System A (54) and System N (55). Accordingly, our metabolomics data demonstrate that i.v. supplemented asparagine reaches the liver within 30 minutes. The use of asparagine supplementation in the clinic would not be unprecedented, as patients with inborn errors of metabolism affecting the TCA cycle, such as patients with pyruvate carboxylase deficiency, see their symptoms improved when given large quantities of asparagine supplement (56, 57). The metabolically compromised cells from such patients are highly susceptible to apoptosis, and it is believed that the use of asparagine in large quantities directly suppresses apoptosis in these cells (56, 57).

Extracellular depletion of asparagine using bacterial L-asparaginase (ASNase) has been successfully used as a chemotherapeutic treatment of pediatric acute lymphoblastic leukemia (ALL) (58). Leukemic cells lack constitutive expression of ASNS, making them auxotrophic for asparagine and highly susceptible to apoptosis when deprived of this amino acid (59). Although ASNase is commonly used for treating this hematological cancer, it should be noted that this chemotherapy can have serious complications, particularly for the liver (60, 61). Our results suggest that the hepatic toxicity of ASNase might be due to the relatively low levels of ASNS in normal livers and that compounds able to boost hepatic ASNS expression could relieve some of these adverse effects.

Numerous studies have placed ASNS at the center of cellular responses to amino acid deprivation and other forms of cellular stress (reviewed in ref. 38). The ASNS gene is a transcriptional target of 2 signaling pathways aimed to ensure cell survival under conditions of imbalanced amino acid availability through the AAR (37) and of increased ER stress through the UPR (23). Through the activation of the general control nonderepressible 2 (GCN2) and the PERK kinases, respectively, these 2 stress-response pathways converge on the phosphorylation of the α-subunit of the initiation factor eIF2, which provokes the attenuation of global protein synthesis and, paradoxically, the preferential translation of a selected population of mRNAs, including the transcription factor ATF4 (38). ATF4 is the primary factor for *Asns* induction and operates as a transactivator through its binding to an enhancer element within the *Asns* promoter (38). The cellular stress induced by CCl_4_ also converges on *Asns* induction but through a different mechanism, independent of ATF4, as evidenced by the fact that this toxin potently blunts ATF4 protein levels (Figure 4A, Figure 5A, and Figure 6C, Supplemental Figure 6B, and Supplemental Figure 7C). Instead, we showed that acute liver injury stimulates the recruitment of LRH-1 to the *Asns* promoter and that this nuclear receptor regulates its transcriptional induction following CCl_4_ injection. This could be the result of either signaling cascades that activate LRH-1 or its coregulators or increased production of an endogenous LRH-1 agonist. In this regard, experiments with *Lrh-1^K289R^* mice are informative as these animals express a mutant form of LRH-1 that cannot be SUMOylated and is viewed as a selective gain-of-function of this nuclear receptor (41, 42). Pericentral *Lrh-1^K289R^* hepatocytes express much higher ASNS levels and are genetically protected from necrosis during acute liver injury. These data raise the interesting possibility that stress pathways could regulate LRH-1 posttranslational modification and activation, although the precise mechanisms remain to be defined. Development of LRH-1 SUMO-specific antibodies and future studies will be needed to characterize posttranslational control of LRH-1 in a variety of contexts, including toxin-induced cellular stress. Finally, the ligand responsiveness of LRH-1 (43, 62, 63) suggests that it could become a therapeutic target in hepatic disorders where amplifying ASNS levels would be beneficial, as during acute liver injury.

## Methods

### Animal experiments.

*Asns^lox/lox^* mice (MGI: 4441742, Supplemental Figure 2A) were crossed with Albumin-Cre mice (B6.Cg-*Speer6-ps1Tg^(Alb–cre)21Mgn^*/J, JAX, Strain 003574) to generate hepatocyte-specific knockout mice (*Asns^hep–/–^* or *Asns^hep+/+^*). Albumin-Cre mice were also crossed with *Lrh-1^lox/lox^* and *Shp^lox/lox^* mice to generate hepatocyte-specific knockout mice (*Lrh-1^hep–/–^* and *Shp^hep–/–^*) separately (40, 47). The generation of the *Lrh-1^K289R^* mouse model is described in a previous study (42). The genetic background of these mouse lines are 90% C57BL/6J mixed with 10% C57BL/6N for *Asns^hep–/–^* mice and pure C57BL/6J for *Lrh-1^hep–/–^*, *Lrh-1^K289R^* and *Shp^hep–/–^* mice. All animals used in this study had free access to food (chow diet, SAFE 150) and water, and kept under normal housing conditions. For CCl_4_ treatment, male mice were i.p. injected either with corn oil (vehicle[veh]) or CCl_4_ (1 mL/kg, Sigma-Aldrich). For the rescue experiments, mice received 2 i.v. injections either with PBS or 240 mg/kg of asparagine (Sigma-Aldrich), glutamate (Sigma-Aldrich), or valine (Sigma-Aldrich) 1 and 8 hours after CCl_4_. For APAP injection, male mice were i.p. injected either with PBS (vehicle [veh]) or APAP (300 mg/kg, Sigma-Aldrich). Mice were not fasted before and during APAP treatment or fasted overnight before APAP treatment. For the rescue experiment for APAP model, mice received 2 i.v. injections either with PBS or 240 mg/kg asparagine 1 and 8 hours after APAP exposure. Mice were sacrificed at indicated times; blood was collected by cardiac puncture, and livers were harvested. Blood samples were centrifuged at 2,000*g* for 10 minutes to obtain serum, which was further used to measure the activity of ALT with a kit from Sigma-Aldrich. Mouse livers were fixed in 4% formalin and paraffin embedded. Samples were sectioned at 4 μm for H&E staining.

### FACS of hepatocytes.

Hepatocytes were isolated from mice as described previously, with minor modifications (40). Isolated cells were first cleaned with Percoll (Sigma-Aldrich) to remove dead cells, then fixed and permeabilized with a solution containing 4% paraformaldehyde and 0.1% Saponin at 4°C for 30 minutes. Next, cells were incubated with GLUL antibody (1:1,000, Sigma-Aldrich, catalog G2781) for 45 minutes, followed by Alexa Fluor 647–conjugated secondary antibody (Thermo Fisher Scientific, catalog A-31573) for 30 minutes at 4°C. In the end, cells were resuspended in 5% EDTA buffer and sorted by FACSAria Fusion sorter (BD Biosciences).

### IHC and Immunofluorescence.

Paraffin-embedded sections of livers were used for IHC and immunofluorescence experiments. Four μm thick sections were dewaxed, rehydrated, and quenched with 3% H_2_O_2_, followed by heat-induced epitope retrieval in 10 mM citrate buffer (pH 6) at 95°C for 20 minutes. Nonspecific antigens were blocked with 1% BSA (Sigma-Aldrich, catalog A7906). Antibodies against p-H2A.X (Ser139) (Cell Signaling, catalog 2577), GLUL (Sigma-Aldrich, catalog G2781), ASNS (Santa Cruz Biotechnology, catalog sc-365809), and 4-HNE (R&D Systems, catalog MAB3249) were incubated overnight at 4°C. Alexa Fluor or HRP-conjugated secondary antibodies were incubated for 1 hour at room temperature. For IHC staining, samples were incubated with DAB followed by nuclear staining using Mayer’s hematoxylin. For immunofluorescence, DAPI was incubated for 10 minutes for nuclear staining.

### TUNEL assay.

A TUNEL assay kit (Promega, catalog G3250) was used to detect DNA damage, according to the manufacturer’s instructions. After TUNEL labeling, DAPI was incubated for 10 minutes to stain the nuclei.

### Amino acid measurement.

For the amino acid measurement after Asn supplementation, C57BL/6J mice received 1 i.v. injection either with PBS or asparagine 1 hour after CCl_4_ and were sacrificed 30 minutes after i.v. injection. The livers were collected and snap-frozen immediately. The amino acid profiling was carried out as previously described (53) using the HILIC-based HRMS method.

### Cell culture, transient transfection, and luciferase assay.

HEK293T cells were cultured in DMEM 4.5 g/L glucose (Gibco) supplemented with 10% FBS, and 1% nonessential amino acid (Gibco) in a humidified incubator with 5% CO_2_ at 37°C. For luciferase assay, HEK293T cells were cotransfected with pGL4-TK reporter constructs driven by the *Asns* promoter consisting of several WT or mutated LRH-1 response elements, in the presence of either pCMV-empty control (EV) or pCMV-LRH-1 constructs using BioT transfection reagent (Bioland Scientific LLC) according to the manufacturer’s instructions. Luciferase activities were measured 24 hours after transfection (Promega) following the manufacturer’s protocol.

### Western blotting.

Total liver lysates were prepared using 50 mg of liver tissue lysed in lysis buffer by sonication (50 mM Tris [pH 7.4], 150 mM NaCl, 5 mM EDTA, 1% NP-40, 0.1% SDS, 0.5% sodium deoxycholate, and protease and phosphatase inhibitors). For chromatin fractions, 50 mg of liver tissue was incubated in Tampon A buffer (10 mM HEPES [pH 7.4], 10 mM KCl, 1.5 mM MgCl_2_, 0.5 mM DTT, and protease and phosphatase inhibitors) and were lysed with a Dounce homogenizer. The homogenized lysates were then passed through 25 G needles 8 times and centrifuged at 1,376*g* for 5 minutes at 4°C to obtain the nuclear fraction. The pellets were incubated in Tampon B buffer (50 mM Tris [pH 7.4], 150 mM NaCl, 0.1% Triton-X100, 1% NP-40, and protease and phosphatase inhibitors) for 30 minutes on ice. After centrifugation at 2,151*g* for 5 minutes, the remaining insoluble pellets contained mainly chromatin. The pellets were resuspended in Tampon B buffer and sonicated to extract protein from chromatin. Western blotting was carried out as previously described (64). Antibodies to ASNS (Santa Cruz Biotechnology, catalog sc-365809, 1:500, 0.4 μg/mL), GLUL (Sigma-Aldrich, catalog G2781, 1:10000, 0.7 μg/mL), GLS2 (Abcam, catalog ab113509, 1:1000, 1 μg/mL), p-H2A.X (Cell Signaling, catalog 2577, 1:1000, 13 ng/mL), H2A.X (Santa Cruz Biotechnology, catalog sc-54607, 1:200, 0.5 μg/mL), ATF4 (Cell Signaling, catalog 11815, 1:1000, 68 ng/mL), LRH-1 (R&D Systems, catalog PP-H2325-00, 1:500, 2 μg/mL), HISTONE 3 (Cell Signaling, catalog 9715, 1:1000, 9 ng/mL), TUBULIN (Sigma-Aldrich, catalog T5168, 1:4000, 1.25 μg/mL), and HSP90 (Santa Cruz Biotechnology, catalog sc-101494, 1:500, 0.4 μg/mL) were used for blotting. See complete unedited blots in supplemental material.

### qRT-PCR.

For FACS-sorted hepatocytes, RNA was extracted by an optimized method called MARIS, or method for analyzing RNA following intracellular sorting (65), which used a column-based extraction method to generate RNA with high quality for transcriptome profiling from fixed and sorted cells. For livers, RNA was extracted using TRIzol (Roche) and reverse transcribed to complementary DNA using QuantiTect Reverse Transcription Kit (Qiagen) following the manufacturer’s protocol. Expression of selected genes was analyzed with the LightCycler 480 System (Roche) and SYBR Green chemistry (Roche). qRT-PCR results were presented relative to the value of housekeeping gene, *Cyclophilin* (ΔΔCT method). Primers for qRT-PCR are listed in Supplemental Table 1.

### Binding site analysis and ChIP-qPCR.

The proximal 1,200 bp sequence upstream of the transcription start site of the *Asns* gene was used for binding site analysis, based on the published binding motif of LRH-1 (50). ChIP analysis was performed as described previously, with minor modifications (41). In brief, freshly isolated livers were homogenized in ice-cold PBS containing protease and phosphatase inhibitors, formaldehyde was added from a 37% stock (v/v) to a final concentration of 1%, and samples were rotated on a shaker for 10 minutes at room temperature followed by the addition of glycine to a final concentration of 0.125 M. The cell pellet, collected by centrifugation (800*g*, 5 minutes at 4°C), was resuspended in buffer A (10 mM HEPES [pH 7.4], 10 mM KCl, 1.5 mM MgCl_2_, 0.5 mM DTT, and protease and phosphatase inhibitors) and homogenized in a Dounce homogenizer to release the nuclei. After centrifugation at 800*g* for 5 minutes at 4°C, the nuclear pellet was resuspended in nuclear lysis buffer (50 mM HEPES [pH 7.5], 150 mM NaCl, 1 mM EDTA, 0.1% sodium deoxycholate, 1% Triton X-100, 0.1% SDS, and protease and phosphatase inhibitors) and was disrupted using the Bioruptor sonication device for 45 minutes with a pulse on 30 seconds and pulse off 30 seconds to shear chromatin. The supernatant was precleaned with Protein A-agarose (Roche) and further used for immunoprecipitation experiments with anti-LRH-1 antibody (R&D Systems, catalog PP-H2325-00, 8 μg/mL) and processed as described previously (41). ChIPed DNA was purified using the PCR Clean-up Extraction Kit (Macherey-Nagel), after which qRT-PCR was performed as described previously (41). Data were normalized to the input (fold differences = 2^– (Ct–sample – Ct–input)^). ChIP primer sequences are listed in Supplemental Table 2. Normal mouse IgG (Santa Cruz Biotechnology, catalog sc-2025) was used as a negative control.

### Human data sets analysis.

Human liver data sets were obtained from Gene Expression Omnibus under the accession number GSE54255 (human liver slices), GSE38941, and GSE28619. Gene differential expression analysis between treatments or patients and controls was performed using GEO2R tool with limma precision weights for each gene set. *P* values were adjusted by Benjamini & Hochberg.

### Statistics.

Data are expressed as mean ± SEM. For experiments with only 2 groups, the unpaired 2-tailed *t* test was used for statistical comparison. 1-way ANOVA with Bonferroni’s posthoc test was used to compare the means of 2 or more independent groups. 2-way analysis of variance with Bonferroni’s posthoc test was used for comparison of magnitude of changes between different treatments from different groups. All statistical analyses were performed in the GraphPad Prism 6.0 software. All *P* values under 0.05 were considered significant.

### Study approval.

All animal experiments were approved by the Veterinary Office of the Canton of Vaud, Switzerland (authorization no. 3520) and performed in accordance with our institutional guidelines.

## Author contributions

YS, HD, and KS conceived and designed the study. YS and HD performed animal experiments. YS and AF performed molecular experiments. FP, AJ, NB, and AP contributed to animal experiments. EY and MK contributed to the immunostaining experiments. YS, HD, and KS analyzed the data and wrote the manuscript.

## Supplementary Material

Supplemental data

## Version 1 01/31/2023

In-Press Preview

## Version 2 04/03/2023

Electronic publication
