# Supplementary material for: Asparagine protects pericentral hepatocytes during acute liver injury
Source: J Clin Invest. 2023 Apr 3;133(7):e163508. doi: 10.1172/JCI163508 (PMC10065070; doi:10.1172/JCI163508)

# Supplemental Figure 1

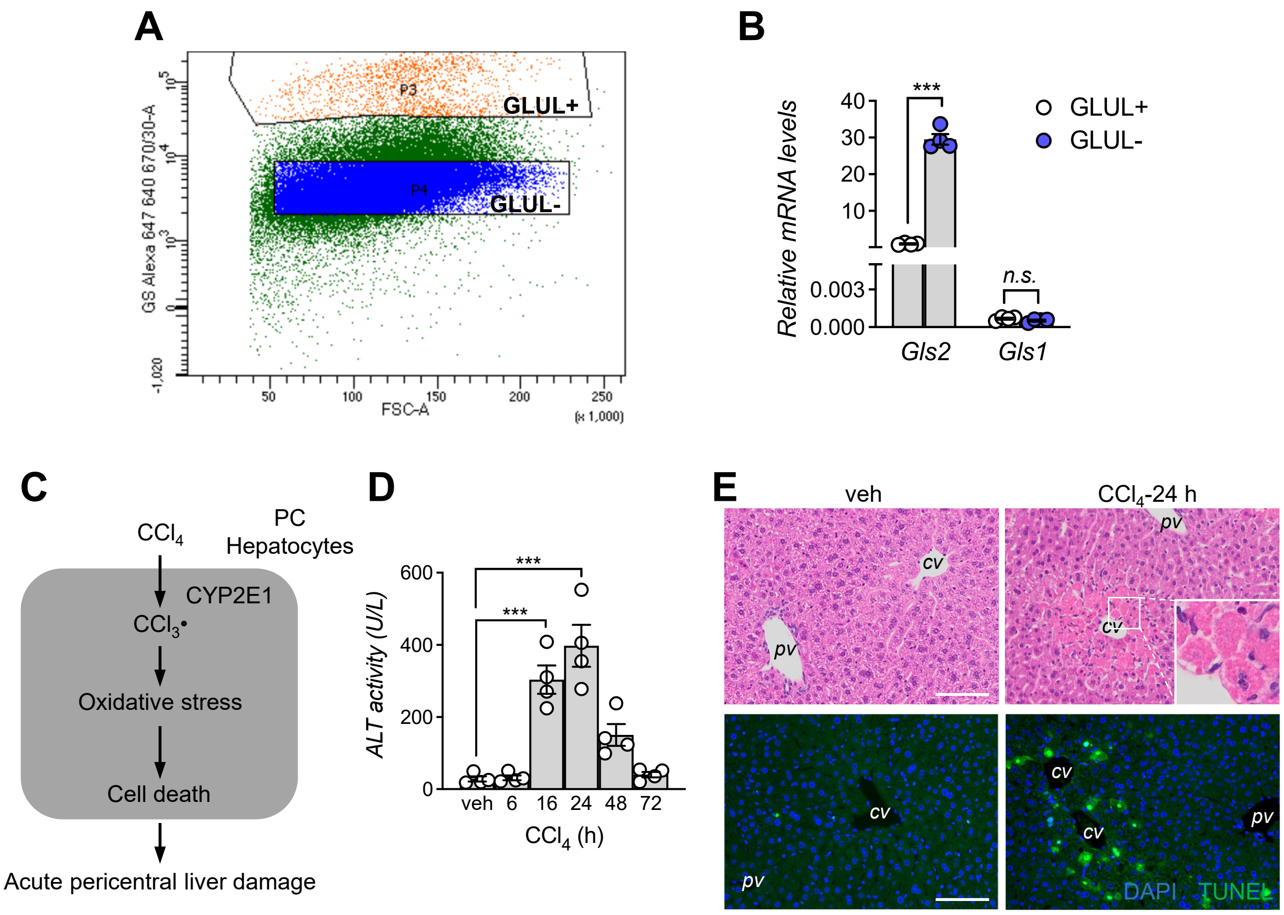


**Supplemental Figure 1. FACS of GLUL+ and GLUL- hepatocytes and $CCl_4$-induced acute pericentral liver injury model.** (A) A representative flow cytometry profile of GLUL-labeled hepatocytes. Hepatocytes in the two squares were collected, cells in P3 polygon representing GLUL+ hepatocytes, and cells in P4 square representing GLUL- hepatocytes. (B) mRNA expression levels of *Gls2* and *Gls* in sorted GLUL+ and GLUL- hepatocytes. n = 4 animals for each group. (C) Scheme showing acute pericentral liver injury induced by $CCl_4$. PC, pericentral. (D) ALT activity in serum from C57BL/6J mice collected at indicated time points after $CCl_4$ injection. n = 4 animals for each group. veh, vehicle. (E) H&E staining and TUNEL assay in livers from mice treated with corn oil (vehicle) or $CCl_4$ for 24 h. cv, central vein. pv, portal vein. Scale bar, 100 μm. Error bars denote SEM. Statistical analysis was performed by one-way ANOVA followed by Bonferroni's post-hoc test (B, D). *n.s.*, not significant, $***P < 0.001$.

## Supplemental Figure 2

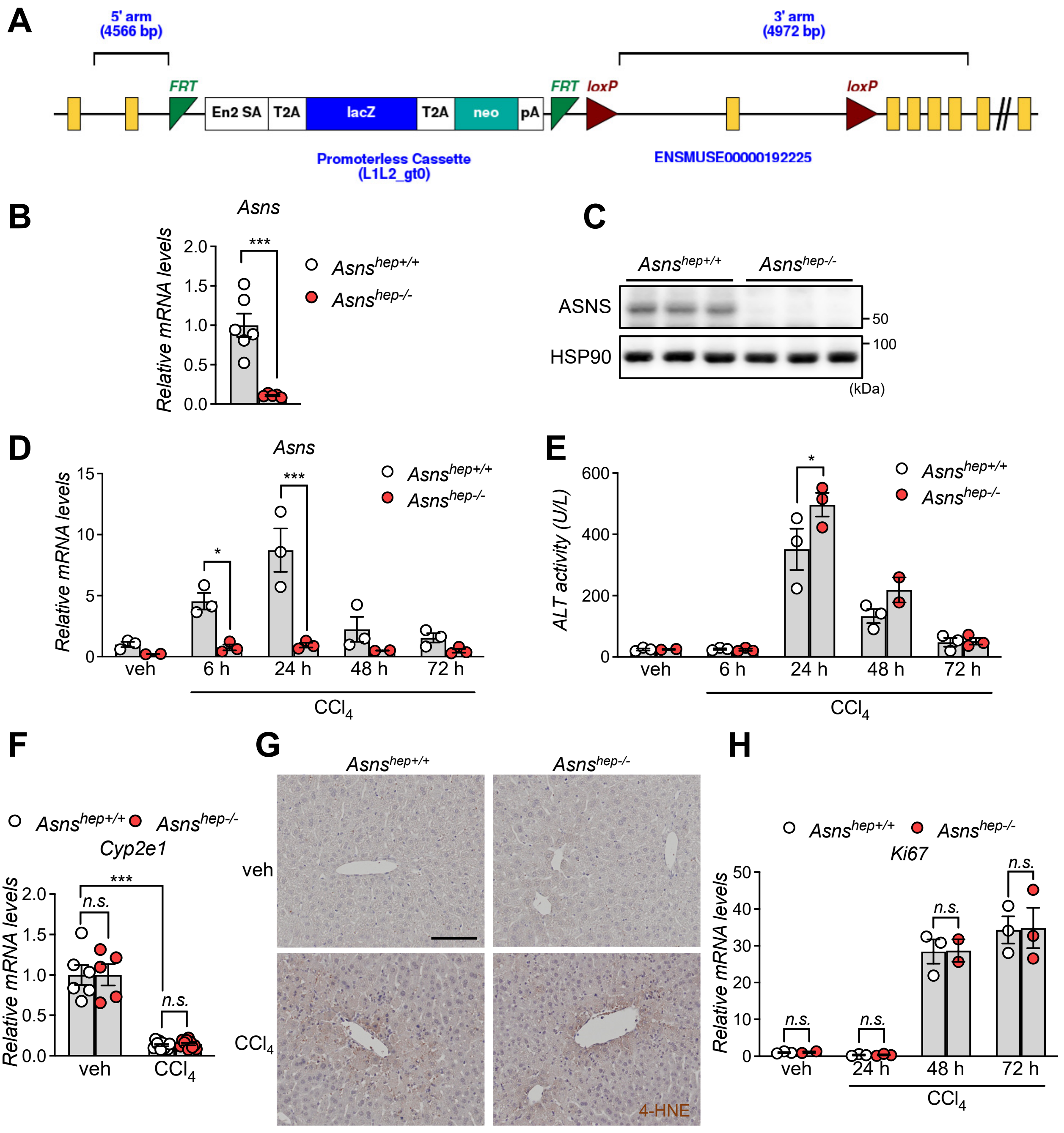


**Supplemental Figure 2. ASNS does not affect *Cyp2e1* expression and lipid peroxidation upon $CCl_4$.** (A) Scheme of *Asns*$^{lox/lox}$ mouse line. loxP sites were inserted up- and down-stream of the targeted exon (exon 3). The L1L2_gt0 cassette was inserted between exon 2 and 3 and is composed of an FRT-flanked lacZ/neomycin sequence. Flp-mediated recombination removed the neo cassette leaving exon 3 floxed. (B-C) mRNA and protein analyses of total liver lysates from *Asns*$^{hep+/+}$ and *Asns*$^{hep-/-}$ mice. n = 6 (*Asns*$^{hep+/+}$) and n = 5 (*Asns*$^{hep-/-}$) animals. (D-E) Hepatic *Asns* expression level and serum ALT activity in *Asns*$^{hep+/+}$ and *Asns*$^{hep-/-}$ mice treated or untreated with $CCl_4$ at indicated time points. n = 3 (all time points of *Asns*$^{hep+/+}$ mice, *Asns*$^{hep-/-}$ 6 h, 24 h, 72h) and n = 2 (*Asns*$^{hep-/-}$ veh, 48 h) animals. (F) mRNA expression level of *Cyp2e1* in livers of *Asns*$^{hep+/+}$ and *Asns*$^{hep-/-}$ mice treated or untreated with $CCl_4$ for 24 h. n = 6 (*Asns*$^{hep+/+}$ veh), n = 5 (*Asns*$^{hep-/-}$ veh) and n = 10 (all other groups) animals. (G) Immunohistochemistry staining of 4-hydroxynonenal (4-HNE) to indicate lipid peroxidation in livers from (F). Scale bar, 100 µm. (H) The expression level of *Ki67* mRNA in livers from (D). Error bars denote SEM. Statistical analysis was performed by unpaired *t*-test (B) and two-way ANOVA followed by Bonferroni's post-hoc test (D-F, H). *n.s.*, not significant, $*P < 0.05$, $***P < 0.001$.

# Supplemental Figure 3

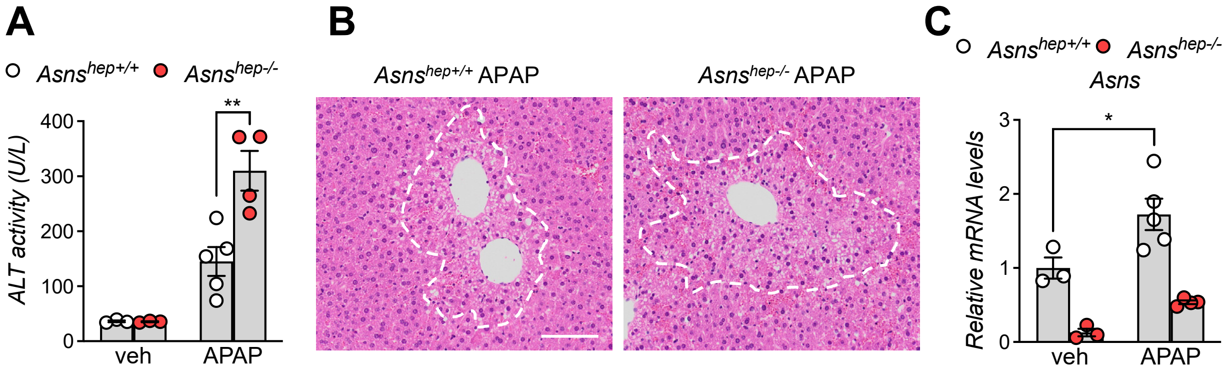


**Supplemental Figure 3. ASNS protects against APAP hepatotoxicity in the fasted state.** (A) Serum ALT activity in overnight-fasted *Asns*$^{hep+/+}$ and *Asns*$^{hep-/-}$ mice treated with vehicle (veh) or APAP for 6 h. n = 3 (*Asns*$^{hep+/+}$ and *Asns*$^{hep-/-}$ veh), n = 5 (*Asns*$^{hep+/+}$ APAP) and n = 4 (*Asns*$^{hep-/-}$ APAP) animals. (B) H&E staining in livers from (A). Injured areas are encircled by white lines. Scale bar, 100 µm. (C) mRNA expression level of *Asns* in livers from (A). Error bars denote SEM. Statistical analysis was performed by two-way ANOVA followed by Bonferroni's post-hoc test (A, C). *$P$ < 0.05, **$P$ < 0.01.

# Supplemental Figure 4

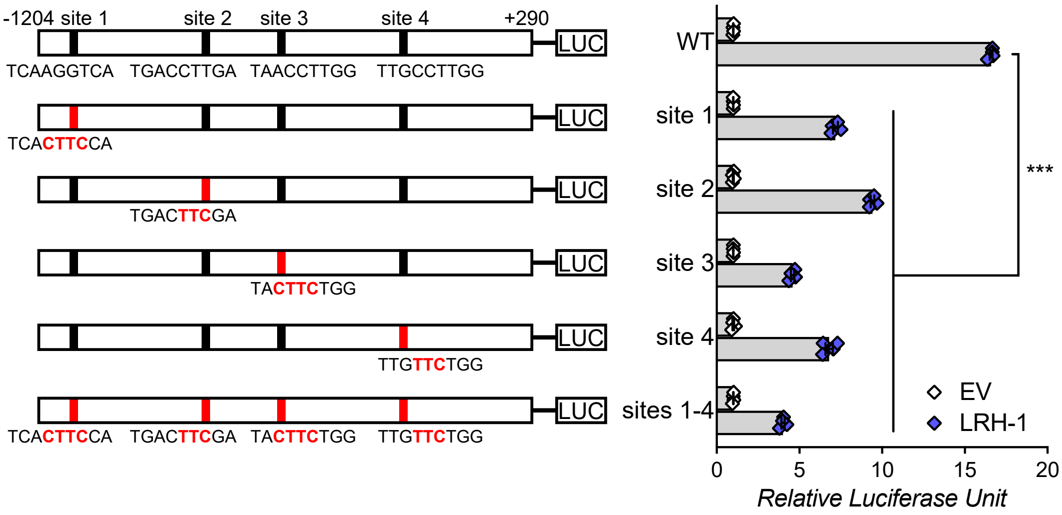


**Supplemental Figure 4. LRH-1 enhances the transcriptional activity of ASNS promoter.** Luciferase reporter activity in HEK293T cells co-transfected with empty vector (EV) or LRH-1 expression construct, and *Asns* promoter-driven luciferase reporters in the absence or presence of the indicated mutations. Bases in red indicate the mutated nucleotides within the promoter. Error bars denote SEM. Statistical analysis was performed by two-way ANOVA followed by Bonferroni's post-hoc test. ***$P < 0.001$.

# Supplemental Figure 5

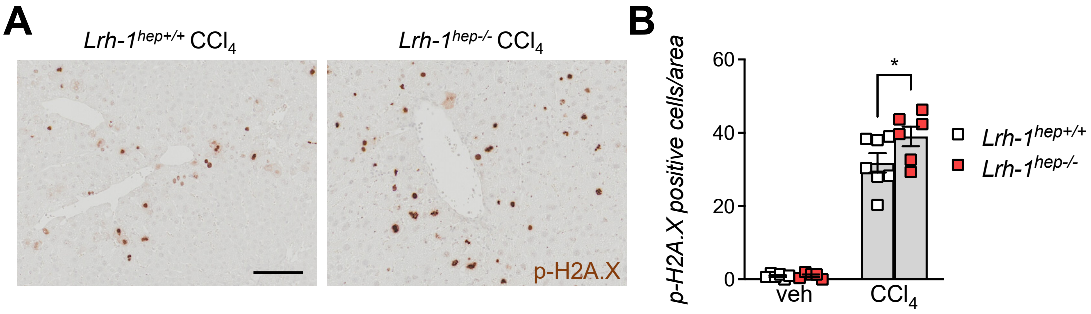


**Supplemental Figure 5. LRH-1 LOF mice show more cell death upon $CCl_4$ challenge. (**A) Representative images of p-H2A.X immunostaining in livers of *Lrh-1*$^{hep+/+}$ and *Lrh-1*$^{hep-/-}$ mice treated or untreated with $CCl_4$ for 24 h. (B) Quantification results of (A). n = 6 (*Lrh-1*$^{hep+/+}$ veh, *Lrh-1*$^{hep-/-}$ $CCl_4$), n = 5 (*Lrh-1*$^{hep-/-}$ veh) and n = 7 (*Lrh-1*$^{hep+/+}$ $CCl_4$) animals. Error bars denote SEM. Statistical analysis was performed by two-way ANOVA followed by Bonferroni's post-hoc test (B). $^*P < 0.05$.

# Supplemental Figure 6

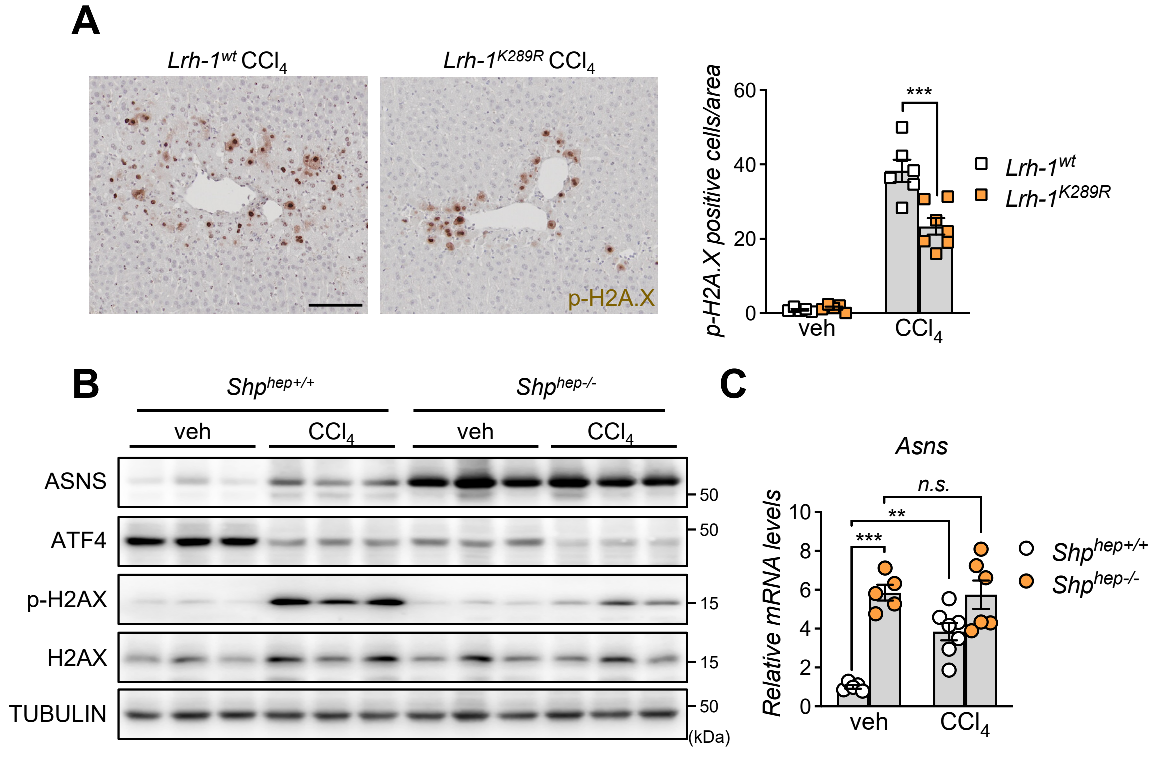


**Supplemental Figure 6. LRH-1 GOF models display less cell death upon $CCl_4$ treatment.** (A) Representative images and quantification results of p-H2A.X staining of livers from *Lrh-1$^{wt}$* and *Lrh-1$^{K289R}$* mice treated 24 h with or without $CCl_4$. Scale bar, 100 µm. n = 5 (*Lrh-1$^{wt}$* veh, *Lrh-1$^{K289R}$* veh), n = 6 (*Lrh-1$^{wt}$* $CCl_4$) and n = 7 (*Lrh-1$^{K289R}$* $CCl_4$) animals. (B-C) Western blotting and mRNA analyses of total liver lysates from *Shp$^{hep+/+}$* and *Shp$^{hep-/-}$* mice treated with or without $CCl_4$ for 24 h. n = 5 (*Shp$^{hep+/+}$* veh, *Shp$^{hep-/-}$* veh), n = 7 (*Shp$^{hep+/+}$* $CCl_4$) and n = 6 (*Shp$^{hep-/-}$* $CCl_4$) animals. Error bars denote SEM. Statistical analysis was performed by two-way ANOVA followed by Bonferroni's post-hoc test (A, C). *n.s.*, not significant, ***P* < 0.01, ****P* < 0.001.

# Supplemental Figure 7

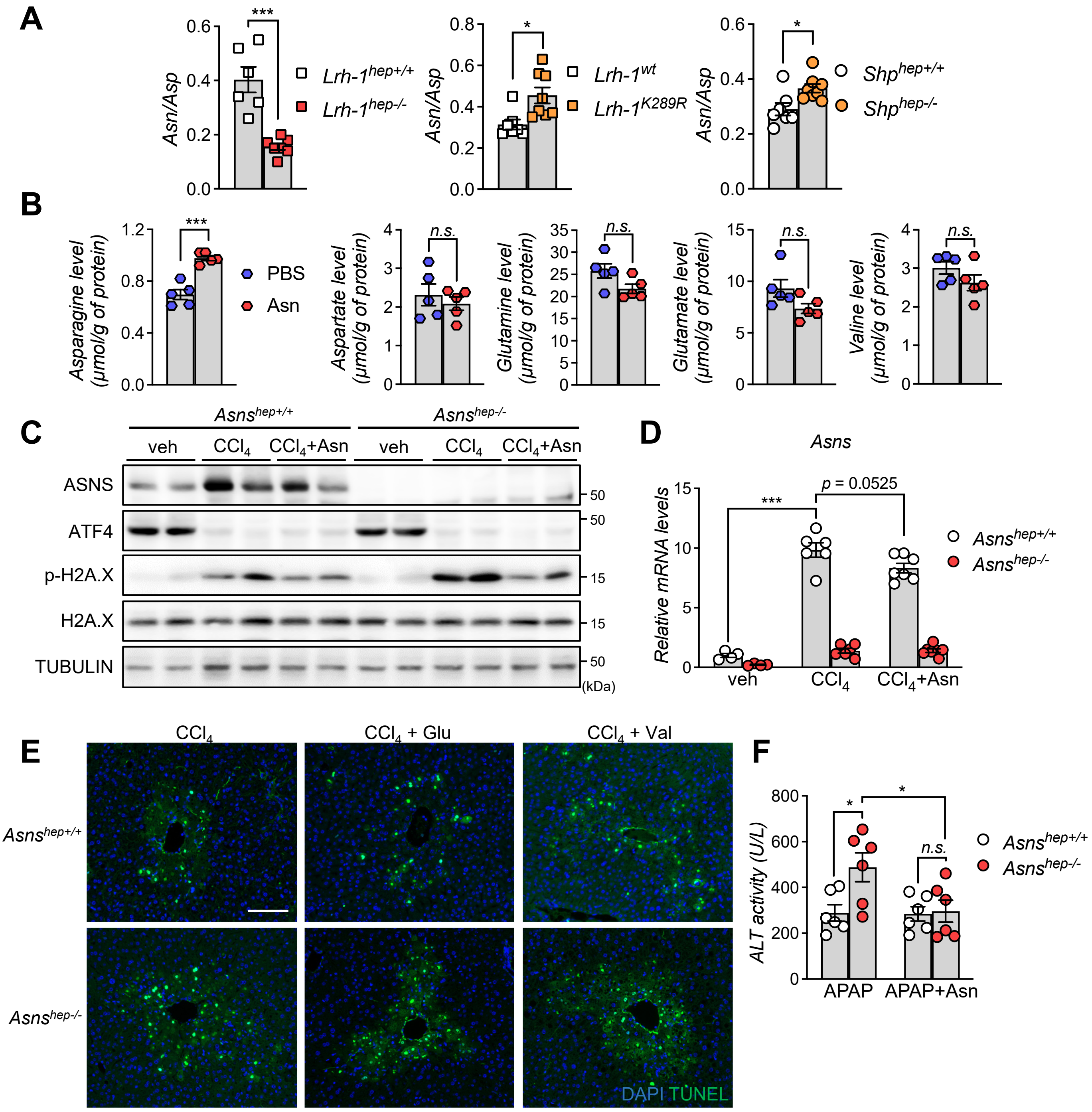


**Supplemental Figure 7. Asparagine mitigates $CCl_4$-induced liver injury in *Asns*$^{hep-/-}$ mice.** (A) The ratio of asparagine (Asn) over aspartate (Asp) in the livers of untreated mouse lines. n = 6 (*Lrh-1*$^{hep+/+}$), n = 7 (*Lrh-1*$^{hep-/-}$, *Lrh-1*$^{wt}$, and *Shp*$^{hep+/+}$) and n = 8 (*Lrh-1*$^{K289R}$, and *Shp*$^{hep-/-}$) animals. (B) The levels of amino acids in the livers of $CCl_4$-treated C57BL/6J mice collected at 30 min after the first i.v supplementation. n = 5 animals for each group. (C-D) Western blotting and mRNA analyses of total liver lysates from *Asns*$^{hep+/+}$ and *Asns*$^{hep-/-}$ mice treated with vehicle (veh), $CCl_4$, or $CCl_4$ followed by Asn injections. n = 4 (veh of *Asns*$^{hep+/+}$ and *Asns*$^{hep-/-}$), n = 6 ($CCl_4$ of *Asns*$^{hep+/+}$ and *Asns*$^{hep-/-}$) and n = 7 ($CCl_4$ + Asn of *Asns*$^{hep+/+}$ and *Asns*$^{hep-/-}$) animals. (E) Representative images of TUNEL assay in livers from $CCl_4$-treated *Asns*$^{hep+/+}$ and *Asns*$^{hep-/-}$ mice, followed by PBS, glutamate (Glu) or valine (Val) injections. (F) ALT activity in serum from APAP-treated *Asns*$^{hep+/+}$ and *Asns*$^{hep-/-}$ mice, followed by Asn injection. n = 6 animals for each group. Error bars denote SEM. Statistical analysis was performed by unpaired *t*-test (A-B) and two-way ANOVA followed by Bonferroni's post-hoc test (D, F). *n.s.*, not significant, *$P$ < 0.05, ***$P$ < 0.001.

**Supplementary Table S1: Sequences of qRT-PCR primers**

| Gene | Forward primer | Reverse primer |
|---|---|---|
| *Glul* | CAAGTGTGTGGAAGAGTTACCTGAGT | TGGCAACAGGATGGAGGTACA |
| *Slc1a2* | CCATTGACTCCCAACACCGA | CGCCAGAGTTACCTTGCACT |
| *Slc1a4* | TGCCGCTGGGTTAAAGGATT | CGTCACAGGGGGTCTCTCTA |
| *Slc1a5* | CACTGCTTTCGGGACCTCTT | CCGATGGGTAGGATGAACCG |
| *Rhbg* | CACTGGGGCAACCACAGTAA | TCAAACTCTCCACGCCAACA |
| *Oat* | ATGTAAGCTCGCTCGTCGTT | ACTGGTCGGATCTGTGGAAC |
| *Asns* | GAGAAACTCTTCCCAGGCTTTG | CAAGCGTTTCTTGATAGCGTTGT |
| *Gls2* | GACCGTGGTGAACCTGCTAT | TGCGGGAATCATAGTCCTTC |
| *Gls1* | AGTGACTTGTGAATCAGCCAG | GTTGCCCATCTTATCCAGAGG |
| *Got1* | GACCATGAGATCCGAACTCA | TGACCAAATACTCGACCTGC |
| *Cyp2e1* | CTTAGGGAAAACCTCCGCAC | GGGACATTCCTGTGTTCCAG |
| *Ki67* | CCTTTGCTGTCCCCGAAGA | GGCTTCTCATCTGTTGCTTCCT |
| *Cyclophilin* | CAGGGGAGATGGCACAGGAG | CGGCTGTCTGTCTTGGTGCTCTCC |

**Supplementary Table S2: Sequences of ChIP-qPCR primers**

| Gene | Forward primer | Reverse primer |
|---|---|---|
| *Asns*-site 1 | AGATGGGTTCACCCTCCAAC | CCACAAGGGATGTACTGCAC |
| *Asns*-site 2 | GTGCATGCATGTGTGTGTGT | AGCACTGTGGAGATGGAAGC |
| *Asns*-site 3 | TCAACTTGATGGAGACTTTGTGA | TCCCTGCAGAGATTAAAAGCA |
| *Asns*-site 4 | CAGAACACCTCCTGGCTCTC | CGGGAAGTTTCATCATGTGG |

## Full unedited gel for Figure 1D

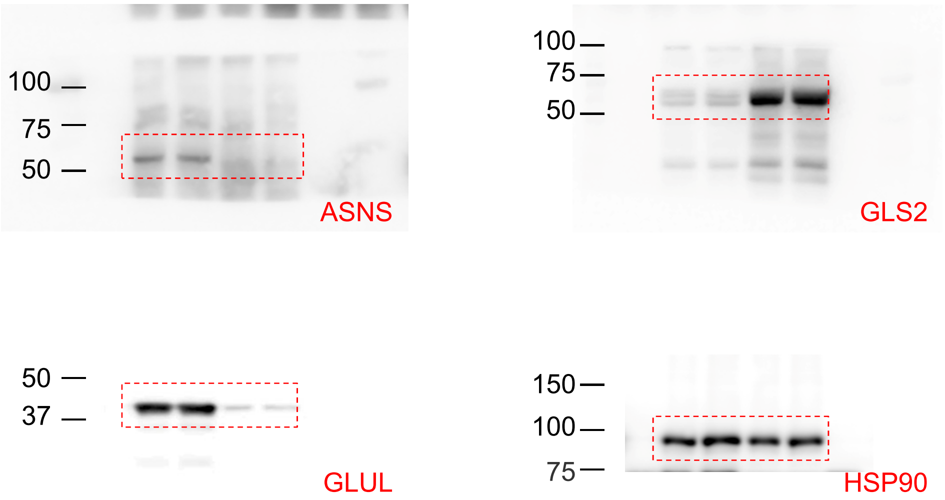


## Full unedited gel for Figure 1E

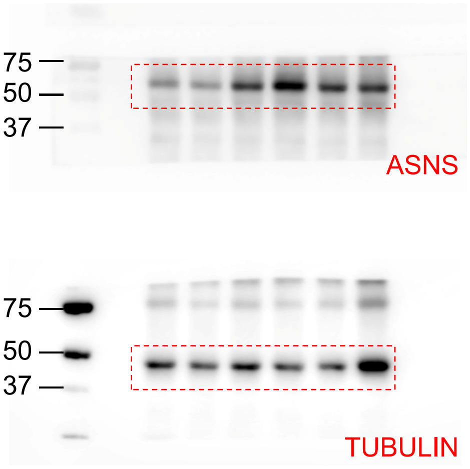

## Full unedited gel for Figure 2B

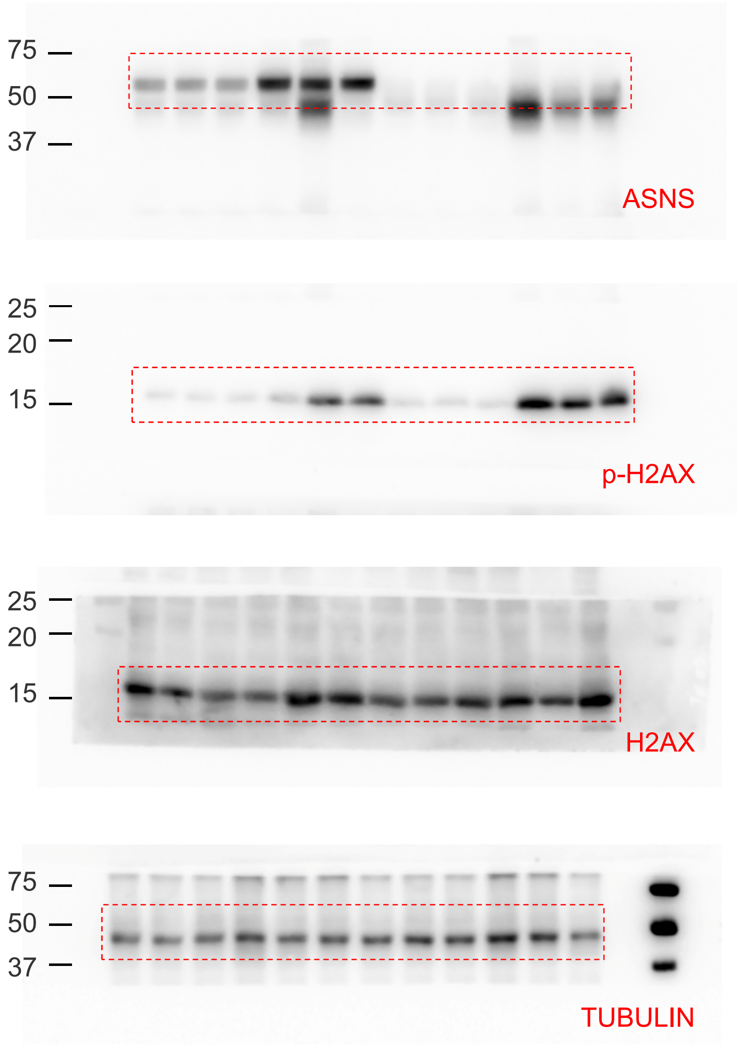

**Full unedited gel for Figure 3E**

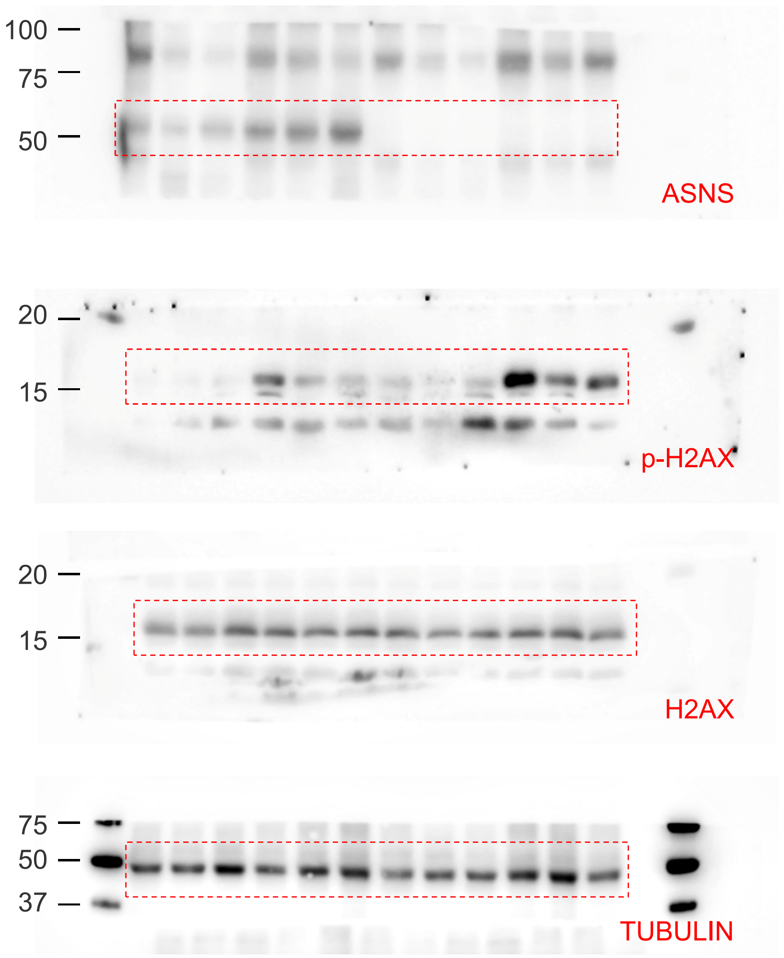

**Full unedited gel for Figure 4A**

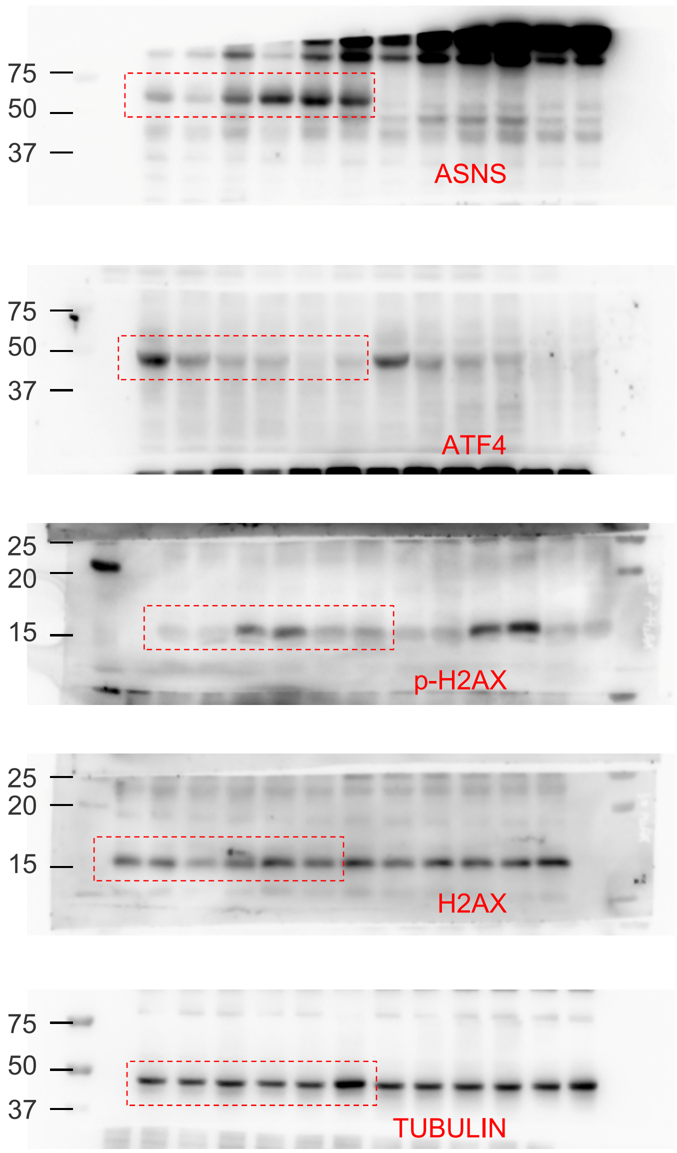

**Full unedited gel for Figure 4C (Left)**

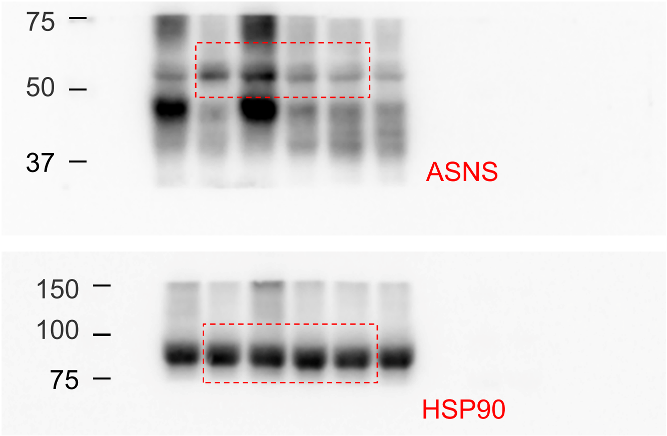


**Full unedited gel for Figure 4C (Right)**

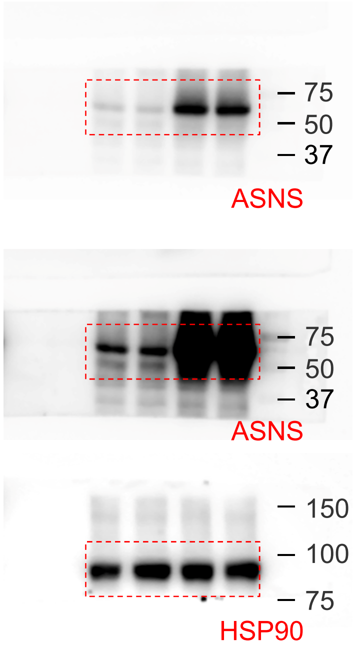

**Full unedited gel for Figure 5A**

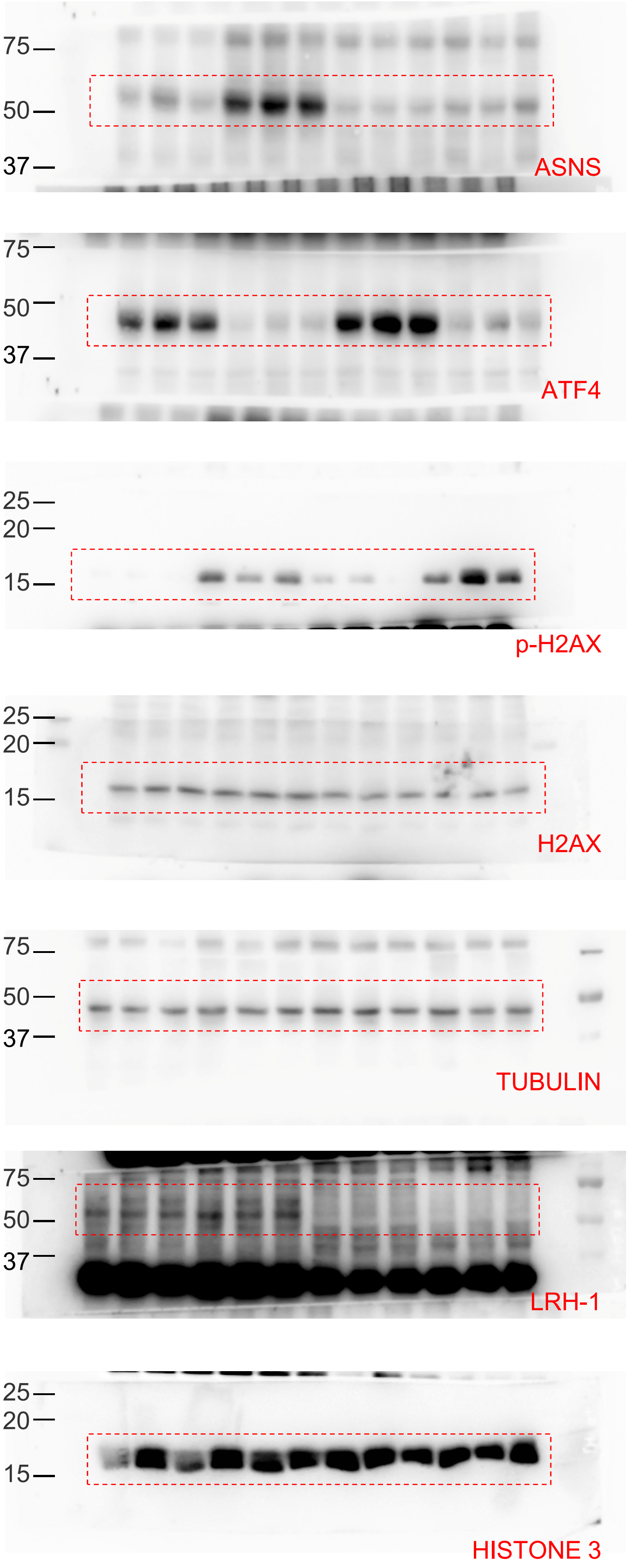

**Full unedited gel for Figure 6C**

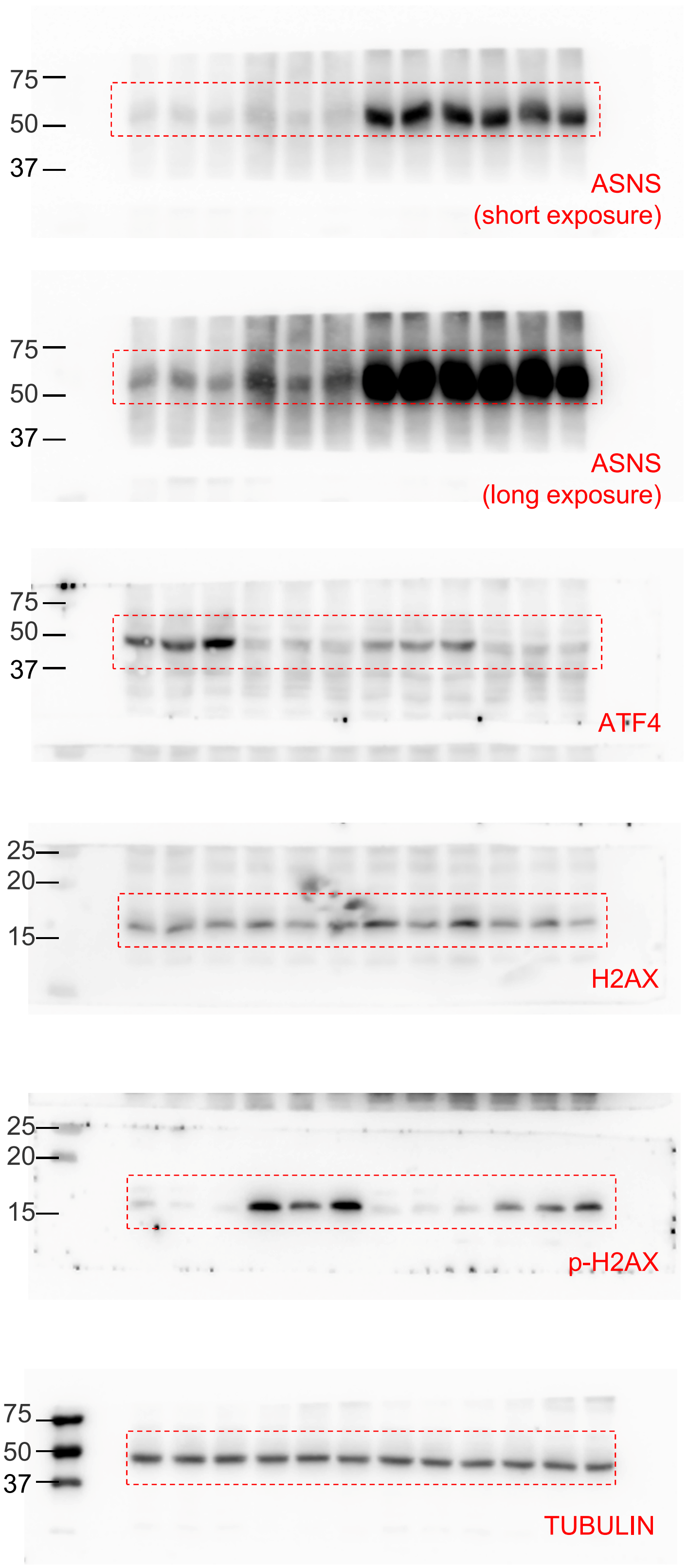

**Full unedited gel for Supplemental Figure 2B**

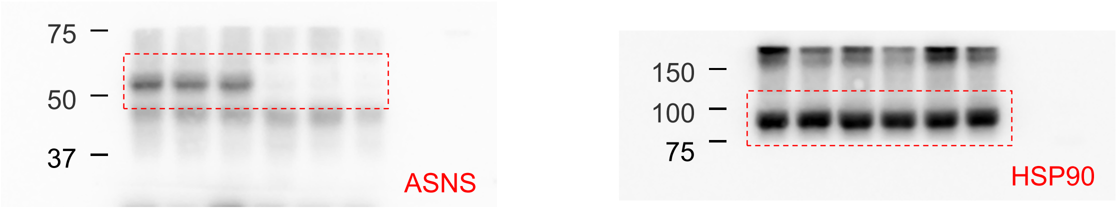


**Full unedited gel for Supplemental Figure 6B**

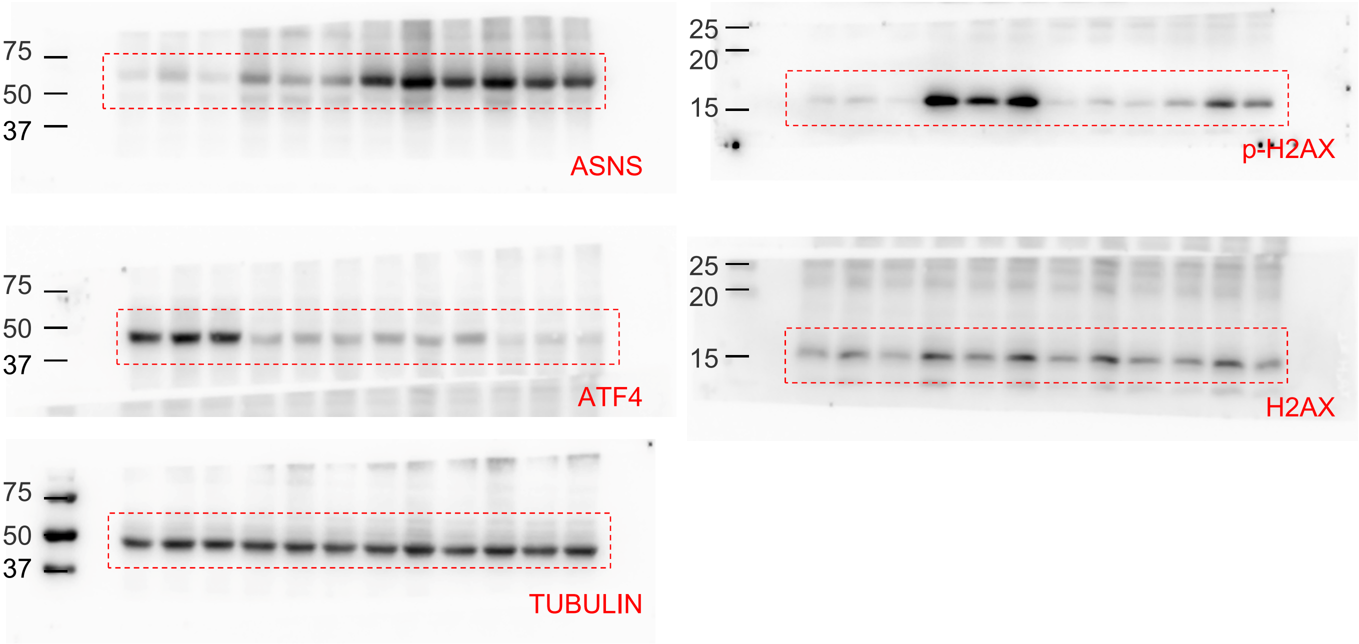

**Full unedited gel for Supplemental Figure 7D**

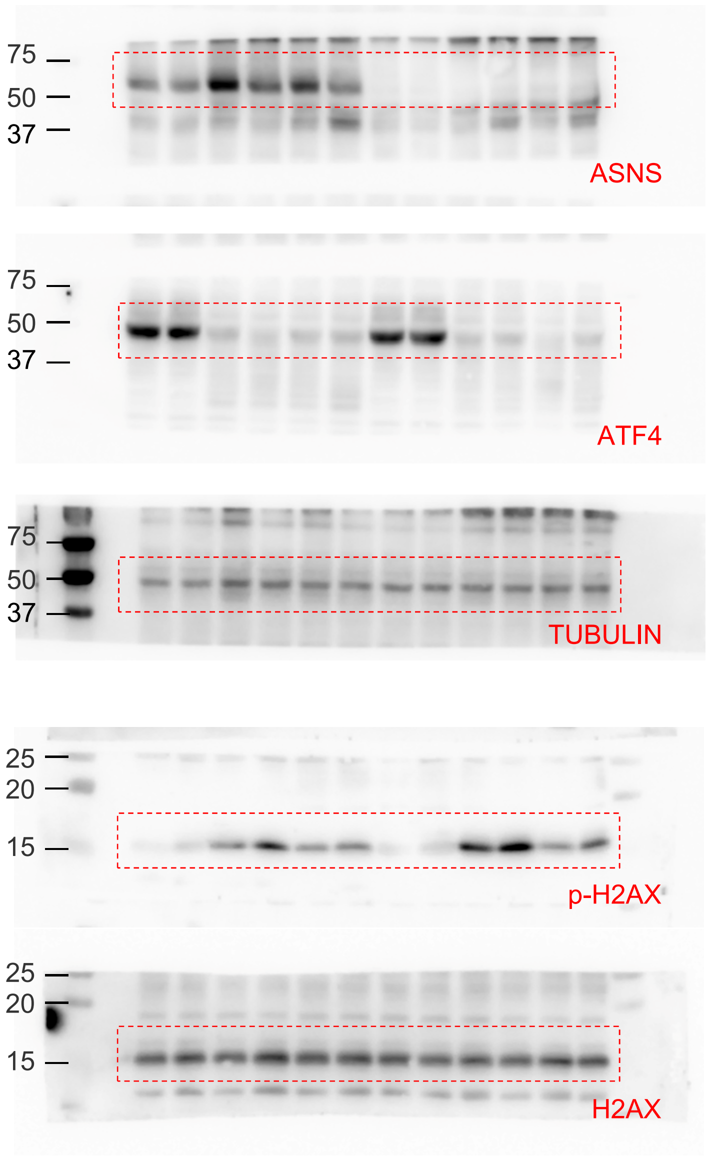

Supplement: Supplemental data [file jci-133-163508-s094.pdf]
